# The Complete Phase Diagram of Monolayers of Enantiomeric *N*-Stearoyl-threonine Mixtures with Preferred Heterochiral Interactions

**DOI:** 10.1021/acs.langmuir.2c01936

**Published:** 2022-10-09

**Authors:** Tetiana Mukhina, Lars Richter, Dieter Vollhardt, Gerald Brezesinski, Emanuel Schneck

**Affiliations:** † Institute for Condensed Matter Physics, Technical University of Darmstadt, Hochschulstraße 8, 64289Darmstadt, Germany; ‡ Max-Planck Institute for Polymer Research, Ackermannweg 10, D-55128Mainz, Germany

## Abstract

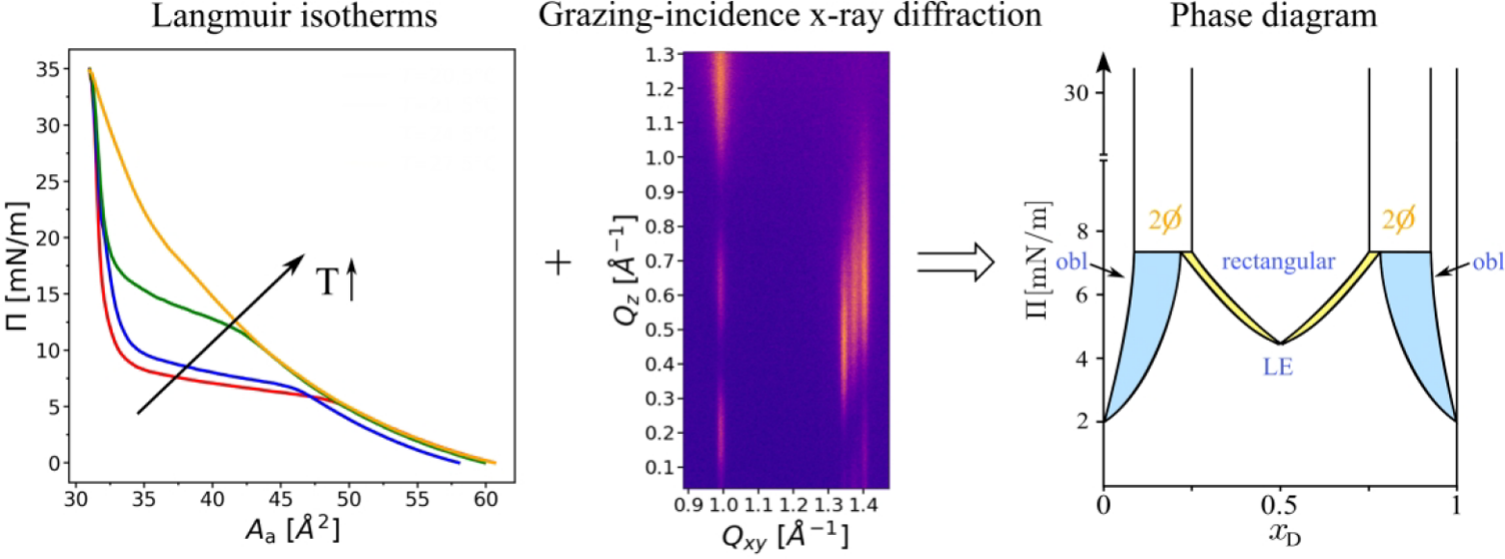

Langmuir monolayers of chiral amphiphiles are well-controlled model systems for the investigation of phenomena related to stereochemistry. Here, we have investigated mixed monolayers of one pair of enantiomers (l and d) of the amino-acid-based amphiphile *N*-stearoyl-threonine. The monolayer characteristics were studied by pressure–area isotherm measurements and grazing incidence X-ray diffraction (GIXD) over a wide range of mixing ratios defined by the d-enantiomer mole fraction *x*_D_. While the isotherms provide insights into thermodynamical aspects, such as transition pressure, compression/decompression hysteresis, and preferential homo- and heterochiral interactions, GIXD reveals the molecular structural arrangements on the Ångström scale. Dominant heterochiral interactions in the racemic mixture lead to compound formation and the appearance of a nonchiral rectangular lattice, although the pure enantiomers form a chiral oblique lattice. Miscibility was found to be limited to mixtures with 0.27 ≲ *x*_D_ ≲ 0.73, as well as to both outer edges (*x*_D_ ≲ 0.08 and *x*_D_ ≳ 0.92). Beyond this range, coexistence of oblique and rectangular lattices occurs, as is clearly seen in the GIXD patterns. Based on the results, a complete phase diagram with two eutectic points at *x*_D_ ≈ 0.25 and *x*_D_ ≈ 0.75 is proposed. Moreover, *N*-stearoyl-threonine was found to have a strong tendency to form a hydrogen-bonding network between the headgroups, which promotes superlattice formation.

## Introduction

Amphiphilic monolayers have been widely used as model systems to mimic the complex physicochemical processes taking place in biological membranes.^1−5^ Monolayer studies of amino-acid-based amphiphiles have been of relevance for numerous applications, such as (bio)sensing, drug delivery, two-dimensional (2D) chiral organization, and recognition,^6−8^ and also for the development of corrosion inhibitors, antibiofouling layers because of their good biodegradability, and low toxicity.^9^ Especially monolayers of amphiphilic *N*-alkanoyl-substituted α-amino acids have been often employed as easy-to-handle models for the investigation of the influence of chirality on the structural organization.^10−15^*N*-alkanoyl-substitution in the amino acid amphiphiles makes them water-insoluble and prevents possible zwitterion formation. The potential of relatively easy production of pure enantiomers facilitated their use as model substances for chiral discrimination studies.^16^ There are many experimental studies on the mesoscopic and microscopic levels based on the relations between the lateral pressure Π and the available area per molecule *A*_a_ (known as pressure–area isotherms),^10−15,17^ on Brewster angle microscopy (BAM),^18−22^ on grazing incidence X-ray diffraction (GIXD), and on infrared reflection–absorption (IRRA) spectroscopy.^23−26^

Recently, thermodynamic and structural studies of enantiomeric and racemic monolayers of *N*-palmitoyl-threonine and *N*-stearoyl-threonine amphiphiles were performed using pressure–area isotherms and GIXD.^27−29^ These studies demonstrated the strong influence of the monolayer composition and intermolecular interactions on the monolayer properties, such as the compression/decompression hysteresis.^27−29^ BAM provided valuable information on the mesoscopic morphology of the condensed phase domains formed in the two-phase coexistence region. Large topological differences in the condensed phase domains of several amino acid amphiphiles were observed and homo- and heterochiral preferences for chiral interaction were discussed. Interestingly, during the growth of racemic *N*-palmitoyl-threonine domains, a crossover was evidenced from preferred homochirality to preferred heterochirality.^27^ GIXD monolayer studies of *N*-alkanoyl-threonine and of *N*-alkanoyl-serine monolayers have shown that the enantiomers form oblique lattice structures at all lateral pressures, whereas the racemates develop orthorhombic structures.^28,29^ The racemate was found to have a much smaller alkyl cross-sectional area in the rectangular lattice structure than the pure enantiomer in the corresponding oblique lattice structure.^29^ The dominant heterochiral interaction in racemic mixtures leads to compound formation with congruent transition pressure, i.e., without change in composition of the involved phases. However, so far little is known about the complete phase diagram in monolayers with compound formation.

To fill this gap, the properties of *N*-stearoyl-threonine-mixed Langmuir monolayers at the air/water interface are investigated in the present work. In particular, the thermodynamic and structural properties of the mixed monlayers are studied at various mixing ratios of the two enantiomeric forms, i.e., *N*-stearoyl-d-threonine and *N*-stearoyl-l-threonine. Pressure–area isotherms were collected for monolayers with d-enantiomer mole fractions of *x*_D_ ∈ (0.5, 1), and the resulting temperature dependence of the phase transition pressures was determined as a function of the sample composition. These measurements allow us to derive the triple-point temperature *T*_0_ at which the LE/LC-transition disappears. GIXD measurements were carried out to probe the miscibility range of l- and d-enantiomers and to investigate the formed lattice structure of condensed monolayers at subnanometer scales. Based on the obtained results, a phase diagram is proposed and discussed.

## Materials and Methods

### Materials

**Figure 1:**
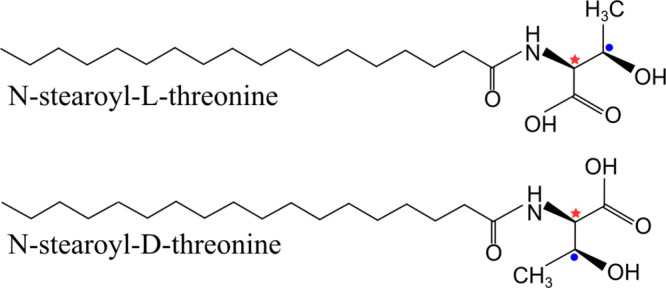
Chemical structures of *N*-stearoyl-l-threonine and *N*-stearoyl-d-threonine amphiphiles. The chiral center of interest is indicated with a red star. The other chiral C atom is indicated with a filled blue circle.

Threonine is an amino acid with two chiral centers (at the 2- and 3-position) and forms four stereoisomers. *N*-stearoyl-l-threonine ((2*S*,3*R*)-2-amino-3-hydroxybutanoic acid) and *N*-stearoyl-d-threonine ((2*R*,3*S*)-2-amino-3-hydroxybutanoic acid) (Figure 1) were synthesized following a protocol established earlier.^16^ Chloroform (purity ≥ 99.9%) and methanol (purity ≥ 99.9%) were purchased from Sigma-Aldrich and used as received. Milli-Q ultrapure water (resistivity = 18.2 MΩ.cm) was obtained from a Milli-Q purification desktop system. Stock solutions of *N*-stearoyl-l-threonine and *N*-stearoyl-d-threonine were obtained by dissolving lipid powder in the mixture of chloroform: methanol = 9:1 (by volume) to a final concentration of ≈1 mg/mL. The corresponding mixtures were prepared from these stock solutions. The subphase was titrated to pH 3 by addition of hydrochloric acid (Merck-Supelco).

### Pressure–Area Isotherm Measurements

Pressure–area isotherms were collected at different temperatures using a Langmuir trough (Accurion, KSV NIMA, Biolin Scientific, Espoo, Finland) equipped with a Wilhelmy paper plate pressure sensor. The trough was temperature-controlled by means of a water circulating thermostat. Before the start of an experiment, 15–20 min were allowed for temperature stabilization of the subphase. The sample solution was spread onto the water surface using a Hamilton syringe. After solvent evaporation (≈ 15 min), the formed monolayers were compressed to the target lateral pressure of 35 mN/m with a constant compression speed of d*A*_a_/d*t* ≈ 2–5 Å^2^/min, where *A*_a_ is the available area per molecule. The data were collected with an accuracy of the surface tension of ±0.1 mN/m and of the molecular area of ± 0.5 Å^2^. Many sources of errors contribute to the uncertainty in the determination of *A*_a_ in an isotherm. Therefore, the crystallographic area per molecule, *A*_c_, determined by GIXD was used to calibrate the isotherms, such that *A*_a_ = *A*_c_ at high lateral pressures. The procedure is clear-cut in cases when only one condensed (LC) phase is present (*x*_D_ = 0.5, 0.6, and 0.7). Under conditions of phase separation (see Figure 6), a linear combination of the coexisting phases LC1 and LC2 should be used. Fortunately, the molecules occupy essentially the same areas in the coexisting phases, so that this more involved procedure is unnecessary in practice.

### GIXD

Grazing-incidence X-ray diffraction (GIXD) experiments were carried out either at the beamline P08 at storage ring PETRA III or at the beamline BW1^30−34^ at storage ring DORIS, both at Deutsches Elektronen-Synchrotron (DESY, Hamburg, Germany). The incident beams had energies of either 15 keV (at P08, corresponding to wavelength λ = 0.826 Å) or 9.5 keV (at BW1, corresponding to wavelength λ = 1.304 Å). In both cases, the beam strikes the air/water interface at a grazing angle of α_i_ = 0.85·α_cr_, where α_cr_ is the critical angle of total external reflection (P08, α_i_ = 0.07°; BW1, α_i_ = 0.13°). In this configuration, only the immediate vicinity of the interface (≈ 80 Å)^35,36^ is probed with an evanescent wave. The footprint of the beam on the water surface was approximately 1 mm × 60 mm at P08 and 2 mm × 50 mm at BW1. A glass plate was placed into the aqueous solution below the beam footprint and ≳ 0.3 mm below the water surface in order to suppress mechanically excited long-wavelength surface waves. The Langmuir trough (R&K, Potsdam, Germany at both P08 and BW1) was enclosed in a sealed helium-filled container and temperature-controlled with a water recirculating thermostat.

The diffraction signal was collected with a one-dimensional position-sensitive detector (PSD) (P08: MYTHEN, PSI, Villigen, Switzerland; BW1: OED-100-M, Braun, Garching, Germany) by scanning the azimuth angle 2θ and, with that, the in-plane component *Q*_*xy*_ = (4π/λ) sin(θ) of the scattering vector . The out-of-plane component, , is encoded in the vertical position of the PSD channels, where α denotes the angle between the scattered direction and the sample plane. At both beamlines the in-plane beam divergence was collimated with a Soller collimator (JJ X-ray, Denmark) placed in front of the PSD providing a full-width-at-half-maximum (fwhm) of Δ_2θ_ ≈ 0.09°, corresponding to  (P08) and *w*_*xy*_^res^ = 0.008 Å^–1^ (BW1), respectively. Further details on GIXD measurements are well described in the literature.^35−40^ The resulting intensity maps *I*(*Q*_*xy*_, *Q*_*z*_) were analyzed by modeling the diffraction peaks with Gaussian functions in *Q*_*z*_-direction and Lorentz functions in *Q*_*xy*_-direction.^36,41^ In the nonlinear least-squares minimization the standard geometrical model constraints concerning peak positions and widths in *Q*_*z*_-direction were imposed.^35,42^ The lattice parameters of the primitive unit cell (lattice parameters *a*, *b*, relative angle γ, in-plane area per chain *A*_*xy*_, cross-sectional area per chain *A*_0_, tilt angle *t*, distortion *d*), as well as the structure of the superlattice formed by whole molecules, were determined from the resulting positions of Bragg peaks and Bragg rods following the procedures described earlier.^35,43,44^

## Results and Discussion

Figure 1 shows the chemical structures of the two stereoisomers *N*-stearoyl-l-threonine and *N*-stearoyl-d-threonine, which differ with regard to the chiral C atom indicated with a red star. The other chiral C atom is indicated with a filled blue circle. l-threonine is an important proteinogenic amino acid, while all other stereoisomers are of minor importance. All measurements were performed with Milli-Q water at pH 3 because at low pH the deprotonation stress on the carboxyl groups is reduced, which in turn stabilizes hydrogen-bond networks (HBN) that involve the charge-neutral state^44^ and reduces undesired influences of electrostatic repulsion or interactions with ions from the subphase.^45^

In the following, we will first discuss isotherm measurements and address the thermodynamic behavior of the monolayers at various temperatures (18 °C ≤ *T* ≤ 28 °C) and sample compositions in terms of the D-enatiomer mole fraction (0.5 ≤ *x*_D_ ≤ 1). Subsequently, we will interpret the obtained GIXD results in terms of the monolayer structure, which allows us to construct a phase diagram.

### Pressure–Area Isotherms

**Figure 2:**
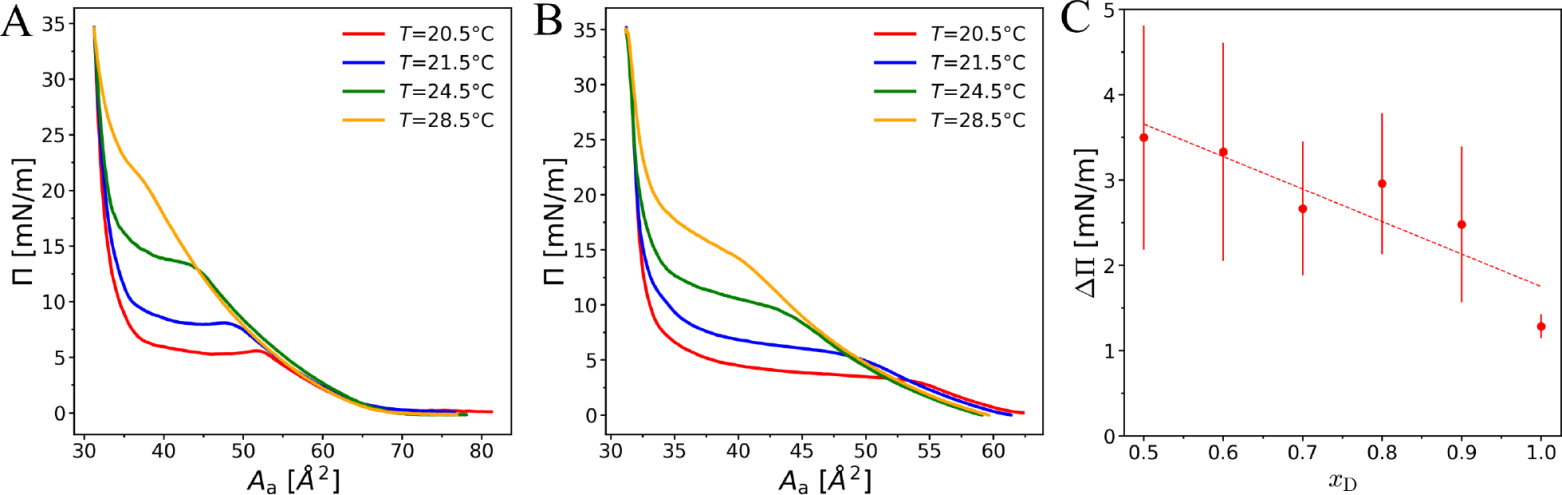
(A and B) Temperature-dependent pressure–area isotherms of an *N*-stearoyl-threonine monolayer with *x*_D_ = 0.9 at compression (A) and at expansion (B). The hysteresis between compression and decompression curves is clearly visible. (C) The difference in transition pressure between compression and expansion, ΔΠ, as a function of *x*_D_. The values were taken at *A*_a_ = 40 Å^2^ and averaged over four temperatures. The error bars are the standard derivation of the data points at different temperatures. The dotted straight line represents a linear fit to the data points.

Figure 2 A and B show representative examples of pressure–area isotherms of the *N*-stearoyl-threonine mixed monolayer with *x*_D_ = 0.9 collected at various temperatures during compression (panel A) and expansion (panel B). The full set of isotherms for all *x*_D_ is shown in the Supporting Information. The isotherms provide information on the phase state of monolayers, as well as the molecular area and thermodynamic characteristics, such as the main phase transition pressure Π_t_ between fluid (LE) and condensed (LC) phases. In contrast to our previous studies^35,44^ we will denote all condensed phases as LC phases in the following, also when they exhibit a molecular superlattice, as discussed further below.

As can be seen in Figure 2, the isotherms exhibit a pronounced plateau indicating LE/LC phase coexistence. Equilibrium isotherms of single component monolayers should have a horizontal plateau. However, in most cases of isotherms presented in the literature, this first-order transition is indicated by a nonhorizontal plateau with slowly rising pressure. Such non-zero slopes have usually been attributed to impurities, nonequilibrium effects, or the preexistence of molecular aggregates or domains.^38,46−48^ For mixtures, the plateau is expected to exhibit a finite slope. In the present case, the transition into the LC phase is characterized by a small hump in the compression isotherms characteristic for a hindered nucleation. This inhibition of nucleation requires supersaturation of the LE phase (overcompression) to initiate the nucleation process. Therefore, the compression curves do not represent equilibrium conditions and, instead, the decompression isotherms, which are better representatives of thermal equilibrium, will be used for further analyses. Above the plateau, a steep increase of the lateral pressure is observed upon further compression, reflecting the low compressibility of the LC phase. As can be seen in Figure 2, the temperature has a significant effect on the shape of the isotherms, in particular on the pressure, extension, and inclination of the transition plateau. This observation holds true for all isotherms collected in this work (see Supporting Information, section S1). At large molecular areas (*A*_a_ ≳ 70 Å^2^, see Figure 2 A), the LE phase coexists with a gas phase. The gas–LE transition is a first-order phase transition with an immeasurably low transition pressure.

#### Hysteresis

All isotherms show a significant hysteresis, i.e., the transition pressure in the compression curves is higher than in the decompression curves. This behavior was practically independent of the temperature in the studied range. A larger discrepancy between compression and decompression cycles suggests that a higher energy barrier must be overcome to initiate the phase transition into the LC phase. Similar observations were reported previously for *N*-stearoyl-serine methyl ester,^19^*N*-stearoyl-tyrosine,^20^ and *N*-stearoyl-allo-threonine.^28^ As also shown earlier,^49^ the hysteresis effect increases with increasing compression rate. Therefore, a comparatively low compression rate was used in the present work. As a quantitative measure the hysteresis, we use the pressure difference ΔΠ at *A*_a_ = 40 Å^2^, which is approximately in the center of the LE/LC phase transition plateau. As seen in Figure 2 C, ΔΠ decreases systematically with increasing *x*_D_ and is minimal at for the pure enantiomer (*x*_D_ = 1). The larger hysteresis for the mixtures can likely be attributed to the longer times required for the diffusive redistribution of the different components in the homogeneously mixed LE phase to reach a critical molecular density for the initiation of the nucleation process.

#### Phase-Transition Pressure

**Figure 3:**
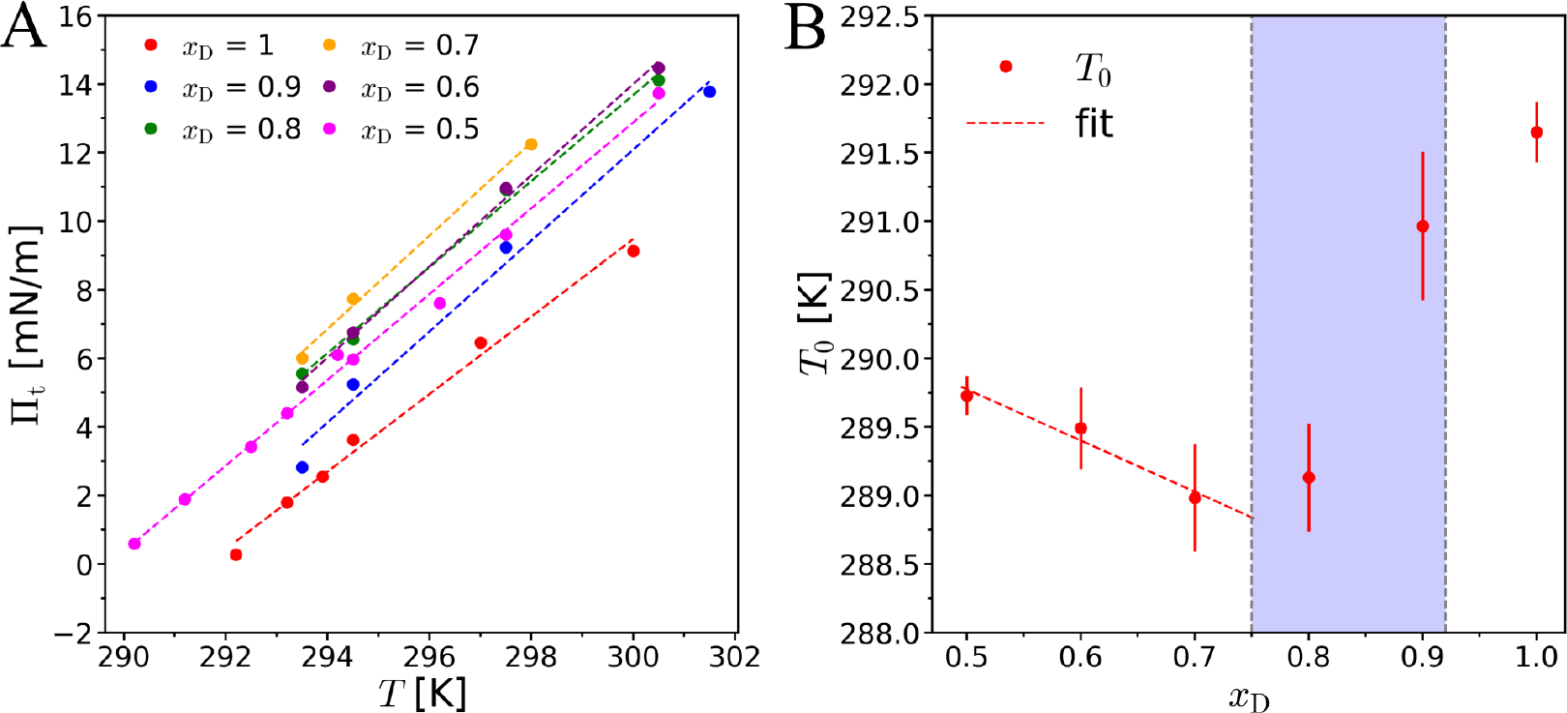
(A) Temperature dependence of the main phase transition pressure Π_t_ of *N*-stearoyl-threonine mixed monolayers for various *x*_D_. Dashed straight lines indicate linear fits to the experimental data points. (B) Characteristic temperature *T*_0_ as a function of *x*_D_. The dashed straight line indicates a linear fit to the first three data points.

The transition pressure Π_t_ was determined for all studied samples following the procedure described in the Supporting Information. In brief, Π_t_ is defined as the onset of the LE/LC phase transition plateau and estimated as intersection point of piecewise linear fits. Figure 3 shows the temperature dependence of Π_t_ for all mixtures investigated. The straight lines superimposed to the experimental data points are linear fits for each sample. The lowest transition pressure is found for the pure d-enantiomer (*x*_D_ = 1) at all measured temperatures, indicating the preferred formation of an LC phase of equal molecules. In contrast, Π_t_ of the racemate (*x*_D_ = 0.5) is always higher than Π_t_ of the pure enantiomer, indicating that the two different enantiomers have to find each other in the liquid phase to form the LC phase nucleus of a heterochiral racemic compound,^27^ which is associated with an entropic barrier. Further addition of d-enantiomers to the racemate leads to a continuous increase of Π_t_ until it reaches its maximum at *x*_D_ = 0.7, followed by a continuous decrease until *x*_D_ = 1 (Figure 3 A). This result is not trivial, considering that the LC monolayer structures of the enantiomer and of the racemate are substantially different (oblique vs. orthorhombic).^27^ The phase transition pressures determined for all studied sample compositions and at several temperatures are summarized in Table 1.

**Table 1:** Variation of the Phase Transition Pressure Π_t_ with Temperature and Sample Composition

	Π_t_[mN/m]			
*x*_D_	*T* = 20.5 °C	*T* = 21. 5 °C	*T* = 24.5 °C	*T* = 27.5 °C
0.5	≈ 4.5	6.0	9.6	13.7
0.6	5.2	6.8	11.0	14.5
0.7	6.0	7.7	≈ 11.6	
0.8	5.6	6.6	10.9	14.1
0.9	2.8	5.3	9.2	≈ 12.8
1.0	≈ 2.0	3.6	≈ 6.7	≈ 10.0

Π_t_ exhibits an almost linear increase with increasing temperature (Figure 3 A) for all sample compositions. The dashed straight lines indicate linear fits to the data points. In the case of one-component systems (pure enantiomer or the compound racemate), the slope dΠ_t_/d*T* is closely related to the transition entropy Δ*S*, which can be calculated with the two-dimensional Clausius–Clapeyron equation.^27^ The extrapolation of the linear fits to Π_t_ = 0 yield a characteristic temperature, *T*_0_.^50,51^ This temperature defines the triple-point at which the gaseous, LE, and LC phases of the monolayer coexist in equilibrium. The higher *T*_0_ the stronger (more favorable) are the intermolecular interactions. The variation of *T*_0_ with the sample composition is plotted in Figure 3 B. The obtained values are summarized in the Supporting Information (Table S7). *T*_0_ of the pure enantiomer is higher than that of the racemate, and the lowest *T*_0_ (the weakest interaction) is in the range of *x*_D_ between 0.7 and 0.8 in accordance with the maximum of Π_t_ in this composition range. Interestingly, the homochiral interactions are stronger than the heterochiral ones, even though the formation of a racemic compound was reported to be preferred over complete enantiomer separation in a certain composition range.^27^

### GIXD

**Figure 4:**
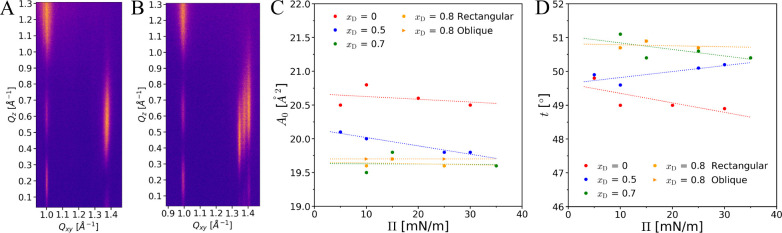
(A) GIXD pattern of an *N*-stearoyl-threonine mixed monolayer with *x*_D_ = 0.7 (Π = 35 mN/m). (B) GIXD pattern of an *N*-stearoyl-threonine mixed monolayer with *x*_D_ = 0.8 (Π = 25 mN/m). (C and D) Pressure dependence of the chain cross-sectional area *A*_0_ (C) and of the tilt angle *t* (D).

To probe the miscibility range and to provide information on the lateral structure of condensed mixed monolayers, GIXD experiments were performed with samples of various enantiomeric compositions. GIXD data of *N*-stearoyl-threonine monolayers with *x*_D_ ∈ {0; 0.5; 1} were recently published.^27^ An oblique lattice (three diffraction peaks) with strongly tilted chains was reported for the enantiomerically pure monolayers, *x*_D_ ∈ {0; 1}, as expected for chiral compounds. The monolayer of the racemate (*x*_D_ = 0.5) featured two diffraction peaks at *Q*_*z*_ ≠ 0, indicating formation of a rectangular lattice structure with chains tilted toward the next nearest neighbor (NNN) direction. This composition-induced transition to an achiral rectangular monolayer structure served as strong evidence of preferred heterochiral interactions between the l- and d-enantiomers, and thus of the formation of an achiral racemic compound. The smaller cross-sectional area *A*_0_ obtained for the racemate demonstrates tighter packing in the racemic monolayer caused by heterochiral interactions (see Figure 4 C).

**Figure 5:**
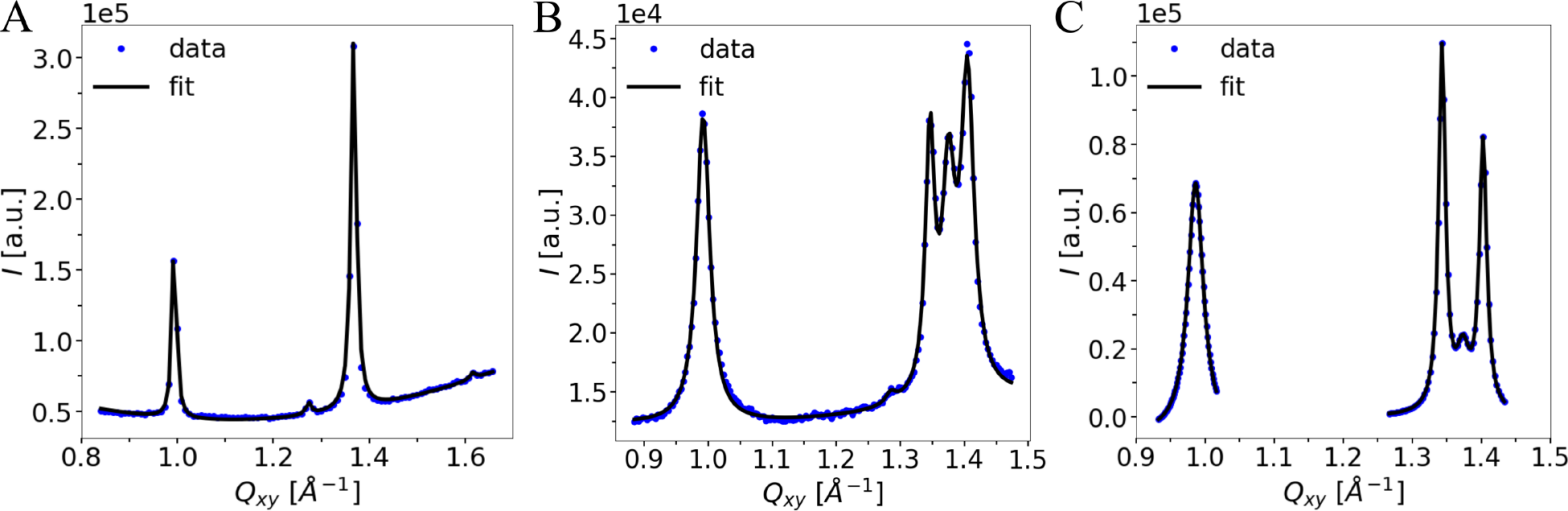
*Q*_*z*_-integrated intensity vs *Q*_*xy*_ (data points) and the corresponding fits (black lines) for an *N*-stearoyl-threonine mixed monolayer with *x*_D_ = 0.5 at Π = 5 mN/m (A), with *x*_D_ = 0.8 at Π = 25 mN/m (B), and with *x*_D_ = 0.9 at Π = 10 mN/m (C).

In the present work, GIXD was used to investigate the monolayer structure in nonracemic mixtures with *x*_D_ ∈ {0.7; 0.8; 0.9}. The diffraction patterns are shown in Figures 4 and 5 and discussed in the following.

#### *N*-Stearoyl-threonine Monolayer with *x*_D_ = 0.7

**Figure 6:**
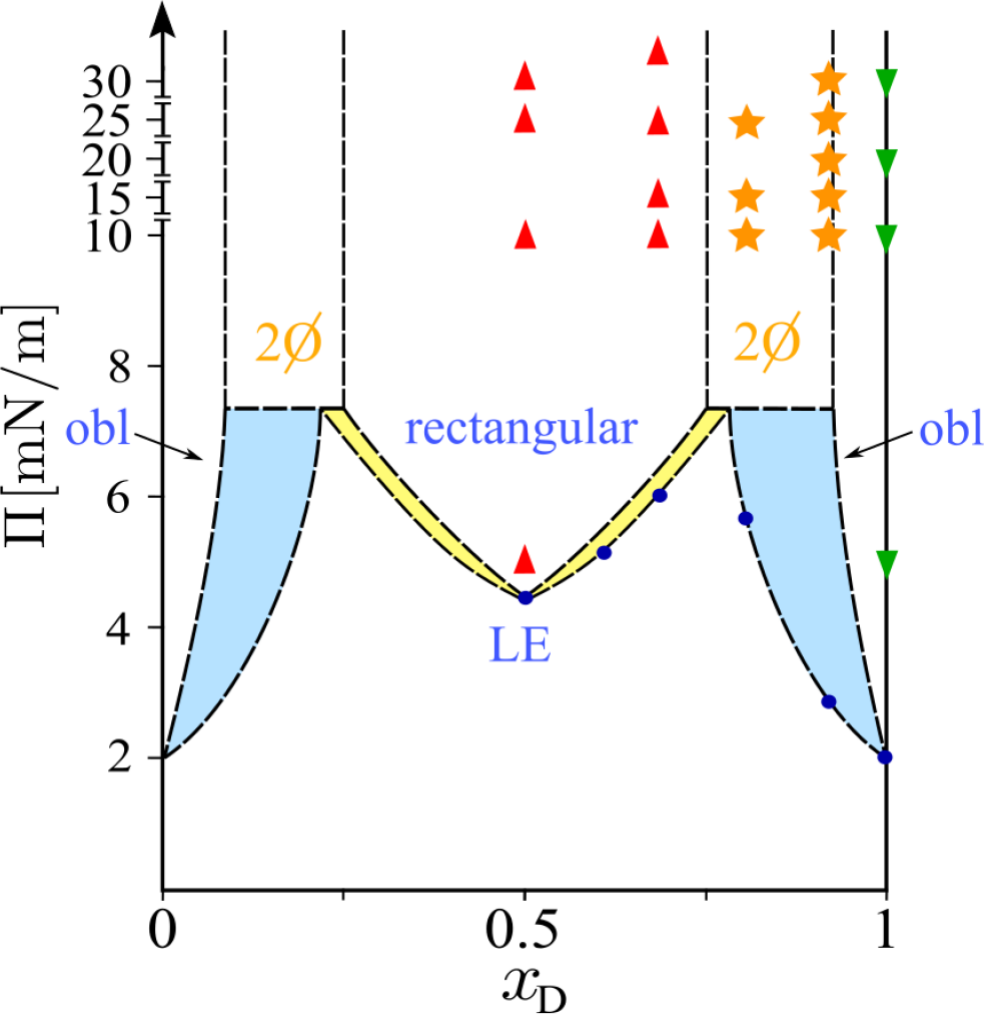
Schematic phase diagram of mixed monolayers of the d- and l-enantiomers of *N*-stearoyl-threonine. The phase transition pressures (Π_t_) determined by isotherm experiments are indicated with solid blue dots. The LC phase of the enantiomers has an oblique lattice structure (denoted as “obl” in the figure), whereas the LC phase of the racemate and of mixtures with 0.27 ≲ *x*_D_ ≲ 0.73 has a rectangular lattice structure. The LC phase in the miscibility gap (0.73 ≲ *x*_D_ ≲ 0.92), denoted as “2Ø” is characterized by the coexistence of rectangular and oblique lattices. The light yellow and light blue areas indicate the coexistence of a disordered LE phase with a rectangular LC phase or an oblique LC phase, respectively. Red upright triangles, green inverted triangles, and orange stars indicate the points where the GIXD data were collected and revealed a rectangular phase, an oblique phase, or phase coexistence, respectively. The diagram has been symmetrically extended to cover the entire range 0 < *x*_D_ < 1.

The diffraction pattern of the monolayer with *x*_D_ = 0.7 features two main peaks at (*Q*_*xy*_, *Q*_*z*_) = (1.374 Å^–1^, 0.604 Å^–1^) and (1.001 Å^–1^, 1.209 Å^–1^) at all pressures investigated (see Figure 4 A). The two weaker intensity maxima at *Q*_*xy*_ = 1.001 Å^–1^, but at lower *Q*_*z*_, can be safely interpreted as satellite fringes of the main reflection, which are expected to occur but often to weak to be observed. The positions and full-widths at half-maximum (fwhm) of the main peaks as well as the reconstructed alkyl chain lattice parameters are summarized in the Supporting Information, Tables S8 and S9. The obtained diffraction pattern is nearly identical with the one obtained for the racemic mixture,^27^ and there is no indication of a possible phase separation. In other words, at *x*_D_ = 0.7 the monolayer forms a rectangular lattice structure with chains tilted toward NNN, as observed for the racemate. However, a small difference in the peak positions is observed, which suggests that the incorporation of additional d-enantiomers to the LC phase of the racemate leads to minor changes in the rectangular lattice structure (see Supporting Information). In fact, the alkyl chain cross-sectional area of *A*_0_ = 19.6 Å^2^ is even slightly smaller than that of the racemate (*A*_0_ = 20.0 Å^2^). The *A*_0_ values as a function of the lateral pressure Π_t_ are plotted for various sample compositions in Figure 4C. The reduced value in the D:L mixture with *x*_D_ = 0.7 may suggest slightly more favorable headgroup interactions in comparison to both the pure enantiomer and the racemic mixture. As observed for those two limiting cases, also for *x*_D_ = 0.7 the tilt angle practically does not depend on the lateral pressure. It is quite large (*t* ≈ 50°) and most probably dictated by the size of the headgroups and by the interactions between them. In addition, due to the small value of the pressure-independent chain cross-sectional area *A*_0_, the chains have to tilt strongly to optimize van der Waals interactions between them. Due to the essentially constant value of tilt angle, the determination of the transition pressure into a nontilted condensed phase via extrapolation^27−29^ is not practicable. The obtained large values of the lattice distortion *d* (see Figure S23 in Supporting Information), which do not exhibit any significant Π dependence, further corroborate the existence of a molecular superlattice. Finally, the additional small but narrow peak at *Q*_*xy*_ ≈ 1.28 Å^–1^, which can be seen only in the *Q*_*z*_-integrated intensity plots of the racemate and the mixtures with *x*_D_ = 0.7 and 0.8 (Figures 6, S17, and S19) is an additional piece of evidence.^35,36^ Based on the available data set, an unambiguous determination of the size of this superlattice cannot be achieved. If we assume the superlattice as an integer multiple of the chain lattice (lattice constants *a* and *b*, angle γ), as is commonly done,^35,44,52^ then the derived molecular superlattice is characterized by the constants *a*_s_ = 2·*a* = 9.78 Å and *b*_s_ = 2·*b* = 13.52 Å. With that, the unit cell of the superlattice has an area of 123.3 *Å*^2^ and accommodates four molecules with an in-plane area of *A*_*xy*_ = 30.8 Å^2^ per molecule (Supporting Information Table S11). The Miller indices (2, −1) and (−2, 1) can be assigned to the small additional peak at 1.28 Å^–1^. Closer inspection of our previously published data on the racemate^27^ reveals the existence of two very weak additional peaks at *Q*_*xy*_ ≈ 1.27 Å^–1^ and *Q*_*xy*_ ≈ 1.62 Å^–1^, corresponding to the peaks with Miller indices (2, −1) and (−2, 1), as well as (2, 1) and (−2, −1), in such a tetra-molecular superlattice (Figure 5 A). The formation of a strong HBN between the headgroups is a plausible explanation of the insensitivity of the lattice structure to the pressure variation and its strong packing stability. Similar structural behavior was observed for selected glycolipids and mixed glycolipid/phospholipid monolayers, which also form a HBN.^35,36^

#### *N*-Stearoyl-threonine Monolayer with *x*_D_ = 0.8

Figure 4 B shows the diffraction pattern collected for a mixed monolayer with *x*_D_ = 0.8. The pattern is more complex and features four distinct diffraction peaks (when disregarding the satellite fringes mentioned before) and is identified as a superposition of two diffraction patterns. One of them originates from a rectangular unit cell, as previously observed for the racemic mixture,^27^ the other one from an oblique unit cell, as was observed for the pure enantiomers. The diffraction peaks at (*Q*_*xy*_, *Q*_*z*_) = (0.992 Å^–1^, 1.212 Å^–1^) and (1.378 Å^–1^, 0.606 Å^–1^) belong to the rectangular lattice and the three peaks at (*Q*_*xy*_, *Q*_*z*_) = (0.992 Å^–1^, 1.212 Å^–1^), (1.347 Å^–1^, 0.490 Å^–1^), and (1.406 Å^–1^, 0.722 Å^–1^) to the oblique lattice, noting that one of the peaks is assigned to both structures. The GIXD data collected at different lateral pressures confirm that the pressure dependence of the structural parameters is negligible, as shown in SI in Figure S18 and Tables S12–S13. In essence, GIXD demonstrates the coexistence of two structures due to phase separation between one phase rich in d-enantiomer (oblique lattice) and one rectangular phase of a mixture between the racemic compound and additional d-enantiomer. The monolayer of the mixture with *x*_D_ = 0.8 is thus in the miscibility gap of the phase diagram, whereas the sample with *x*_D_ = 0.7 forms a homogeneous monolayer. Therefore, one boundary of the miscibility gap must be located between *x*_D_ = 0.7 and *x*_D_ = 0.8. The two coexisting phases possess a chain cross-sectional area of *A*_0_ = 19.6 Å^2^ (rectangular lattice) and *A*_0_ = 19.7 Å^2^ (oblique lattice), which is significantly smaller than that in monolayers of the pure enantiomers (*A*_0_ = 20.5 Å^2^) and of the racemate ( *A*_0_ = 20.0 Å^2^) (see Figure 4 C). This result suggests that adding more d-enantiomer to the racemic compound leads to an even tighter chain packing in the homogeneously mixed phase. Apparently, a quite high fraction of d-enantiomers can be included into the rectangular lattice of the racemate before phase separation occurs. The second phase, which is rich in d-enantiomers, contains only a small fraction of l-enantiomers since the mixture with *x*_D_ = 0.9 is still in the miscibility gap (see Figure 6), but the incorporation also leads to a much tighter packing. The positions of the diffraction peaks of this structure are detectably shifted with respect to those of a pure d-enantiomer monolayer, which confirms that the oblique structure of the d-enantiomer changes immediately due to the incorporation of a quite small fraction of l-enantiomers.

As for monolayers with *x*_D_ = 0.7, at least one additional peak of weak intensity (at *Q*_*xy*_ ≈ 1.29 Å^–1^) can be seen in the *Q*_*z*_-integrated intensities for *x*_D_ = 0.8 (see Figure S19 in Supporting Information). In fact, both phase-separated structures may appear to form molecular lattices by at least 4 molecules (see Table S15 in Supporting Information), but the complexity of the diffraction pattern prevents us from evaluating these superlattices in more detail.

#### *N*-Stearoyl-threonine Monolayer with *x*_D_ = 0.9

The GIXD pattern of an *N*-stearoyl-threonine mixed monolayer with *x*_D_ = 0.9 features three intense Bragg peaks at *Q*_*xy*_ = 0.986 Å^–1^, *Q*_*xy*_ = 1.343 Å^–1^, and *Q*_*xy*_ = 1.403 Å^–1^ (see Figure S20 in the Supporting Information), characterizing an oblique lattice. As observed for the mixture with *x*_D_ = 0.8, the peak positions are shifted with respect to the ones observed with the pure enantiomer (0.978, 1.337, and 1.399 Å^–1^ at 10 mN/m and 10 °C).^27^ The modeled data and the corresponding lattice parameters are presented in Tables S16 and S17 of the Supporting Information. An additional, albeit weaker, peak is visible at *Q*_*xy*_ ≈ 1.373 Å^–1^, indicating phase separation and coexistence of an oblique lattice and a rectangular lattice. The lower intensity of this peak compared with the same peak in the *x*_D_ = 0.8 mixture indicates that the amount of the coexisting rectangular phase is smaller but still detectable (see Figure 5 B, C). Apparently this mixture is closer to the upper boundary of the miscibility gap. Due to significant overlap of peaks from different structures we had to refrain from analyzing peak positions and widths along the *Q*_*z*_-direction, which is why only the *Q*_*xy*_ values are presented in Table S16 of the Supporting Information.

### Phase Diagram

In quasi two-dimensional systems, such as monolayers, the miscibility behavior can be described with phase diagrams as in three-dimensional systems. Complete miscibility or complete immiscibility as well as miscibility gaps can occur. The thermodynamic and structural results described in the previous sections enable us to construct the phase diagram for the studied mixtures. This phase diagram is shown in Figure 6. At *x*_D_ = 0.5, only a single phase with a rectangular lattice structure of the racemic compound is observed. The GIXD results obtained at higher *x*_D_ indicate that a certain amount of the d-enantiomer can be integrated into the rectangular structure of the racemate (up to *x*_D_ ≈ 0.73) to form a homogeneously mixed LC phase above the transition pressure. As shown by the isotherm measurements, the transition pressure at *x*_D_ = 0.7 is the highest, suggesting that higher compression (higher density) is required to overcome the nucleation barrier. The miscibility gap is clearly visible in the GIXD experiments with *x*_D_ = 0.8 and 0.9, where coexistence of rectangular and oblique lattices is observed, of which the latter is comparatively richer in the d-enantiomer. For even higher d fractions (*x*_D_ ≳ 0.92), the remaining l-enantiomer can be completely mixed with the d-enantiomer and forms a single oblique phase. The phase transition pressure of these monophasic mixtures with very high *x*_D_ is lower than that in the miscibility gap (see Figure 6). The proposed phase diagram suggests that a quite large amount of the d-enantiomer can be homogeneously mixed with the racemic compound before phase separation between the orthorhombic (racemic compound) and oblique structures (enantiomers) occurs. The phase diagram is symmetrically composed of practically two diagrams between the racemic compound and the d-enantiomer and correspondingly the l-enantiomer. Here we find two eutectic points at *x*_D_ ≈ 0.75 and *x*_D_ ≈ 0.25. The eutectic mixture is a homogeneous mixture of the racemate and the corresponding enantiomer that undergoes the LE/LC transition at a single pressure that is higher than the transition pressures of the two constituents. At this pressure, the LE state and the two condensed phases (rectangular and oblique) of the eutectic mixture coexist and are in chemical equilibrium. The miscibility gaps in the LC phase are quite large (0.73 ≲ *x*_D_ ≲ 0.92) and (0.08 ≲ *x*_D_ ≲ 0.27).

## Conclusions

We have used pressure–area isotherm measurements and GIXD to investigate monolayers of *N*-stearoyl-threonine with various mixing ratios of its d- and l-enantiomers, defined by the d-fraction *x*_D_. Around the transition between LE and LC phases, all isotherms exhibited a significant compression/expansion hysteresis, which was found to increase systematically in magnitude when going from the pure enantiomer (*x*_D_ = 1) to the racemic mixture (*x*_D_ = 0.5). This behavior can be explained by the longer equilibration times required for the diffusive redistribution of the different enantiomers to form stoichiometric LC structures. At all temperatures, the lowest phase transition pressure, Π_t_, was observed for the pure enantiomer. The racemic mixture *x*_D_ = 0.5 shows the transition at a slightly higher pressure, and the pressure increases systematically with increasing *x*_D_ until the composition limit of a single enantiomerically mixed phase is reached (at *x*_D_ ≈ 0.73) and demixing into two phases with different compositions occurs. Above *x*_D_ ≈ 0.92, Π_t_ decreases again until the value for the pure enantiomer. Interestingly, the triple-point temperature *T*_0_ has a minimum in the miscibility gap, which reflects the least favorable molecular interactions and which is in accordance with the destabilization of the LC phases of the pure enantiomers and the racemic compound by adding the other enantiomer.

The GIXD experiments provided complementary information on the structure of the LC phases. The single “compound” LC phase of the racemic mixture (*x*_D_ = 0.5) is characterized by a rectangular lattice. Our results suggest that in the composition range between *x*_D_ ≈ 0.27 and *x*_D_ ≈ 0.73, additional enantiomers can be integrated into this rectangular lattice, however at the cost of its thermodynamic destabilization, as evidenced by the increase of Π_t_. Between *x*_D_ ≈ 0.73 and *x*_D_ ≈ 0.92, two LC phases coexist, one of which is rectangular while the other one is oblique and resembles that of the pure enantiomer. Based on the combined results of isotherm measurements and GIXD a phase diagram was constructed. This work may thus constitute a significant contribution to our understanding of the mixing and phase behavior of chiral amphiphiles.

## Supporting Information Available

Isotherm measurements of the d:l*N*-stearoyl threonine monolayers at various mixing ratios and temperatures; temperature dependence of the main phase-transition pressure Π_t_ of the mixed d:l*N*-stearoyl threonine monolayers; experimental and simulated GIXD patterns and parameter tables; plots of the pressure dependence of the chain cross-sectional area (*A*_0_), in-plane area per alkyl chain (*A*_*xy*_), tilt angle (*t*), 1/cos(*t*), and distortion (*d*) for the mixed d:l*N*-stearoyl threonine monolayers (PDF)
