# Supplementary material for: The Complete Phase Diagram of Monolayers of Enantiomeric N-Stearoyl-threonine Mixtures with Preferred Heterochiral Interactions
Source: Langmuir. 2022 Oct 9;38(41):12521–9. doi: 10.1021/acs.langmuir.2c01936 (PMC9583611; doi:10.1021/acs.langmuir.2c01936)
Supplement: Supplementary file 1 — la2c01936_si_001.pdf [file la2c01936_si_001.pdf]

The complete phase diagram of monolayers of enantiomeric N-stearoyl-threonine mixtures with preferred heterochiral interactions
Supporting Information

Tetiana Mukhina,† Lars Richter,† Dieter Vollhardt,*,‡ Gerald Brezesinski,† and Emanuel Schneck*,†

†*Institute for Condensed Matter Physics, Technical University of Darmstadt, Hochschulstraße 8, 64289 Darmstadt, Germany*

‡*Max-Planck Institute for Polymer Research, Ackermannweg 10, D-55128 Mainz, Germany*

E-mail: vollhardtd@mpip-mainz.mpg.de; emanuel.schneck@pkm.tu-darmstadt.de

1 Isotherm measurements of the mixed D:L N-stearoyl-threonine monolayers

1.0.1 Isotherm analysis

All collected isotherms were analysed in order to determine the transition pressures Π_t and Π_{LC} at which the transition from LE to LC begins and ends (extension of the coexistence plateau). These values were determined by piece-wise linear fits of the isotherms as demonstrated in Fig. S1. Linear functions were fitted to some parts of LE regions (1) and LC regions (2), as well as a LE/LC coexistence region plateau (3). The intersection points of

the fit of the phase transition plateau (2) with the linear fits of the LE (1) and LC (3) regions define the set of values (A_{LE}, Π_{t}) and (A_{LC}, Π_{LC}), accordingly.

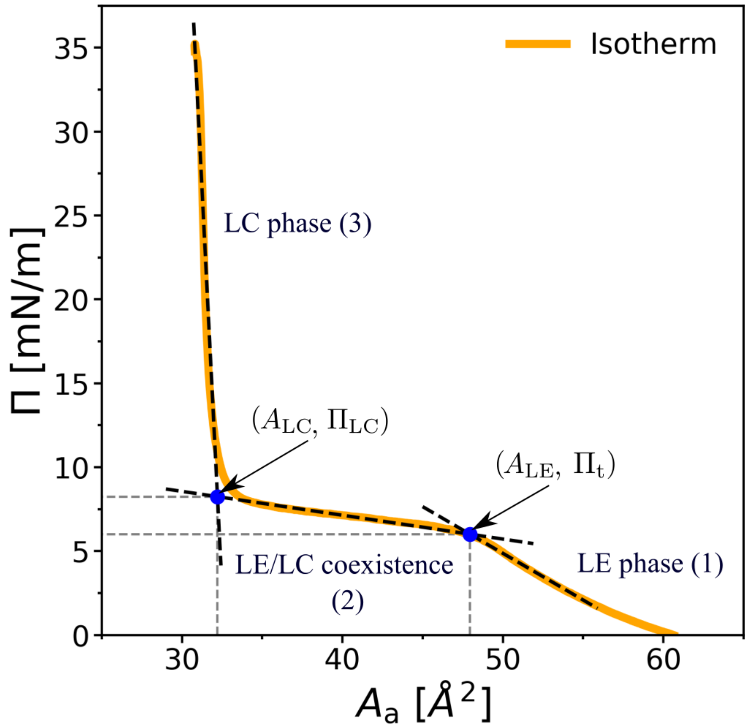

Figure S1: Representative example of the isotherm measurement (orange line) together with the linear fits (black dashed lines) of LE regions (1) and LC regions (2), as well as a LE/LC coexistence region plateau (3). The intersection points of fit lines define the pressures of the onset and of the termination of a LE/LC phase coexistence plateau together with the corresponding available areas per molecule, i.e. (A_{LE}, Π_{t}) and (A_{LC}, Π_{LC}).

1.1 N-stearoyl-D-threonine monolayer ($x_\mathrm{D} = 1$)

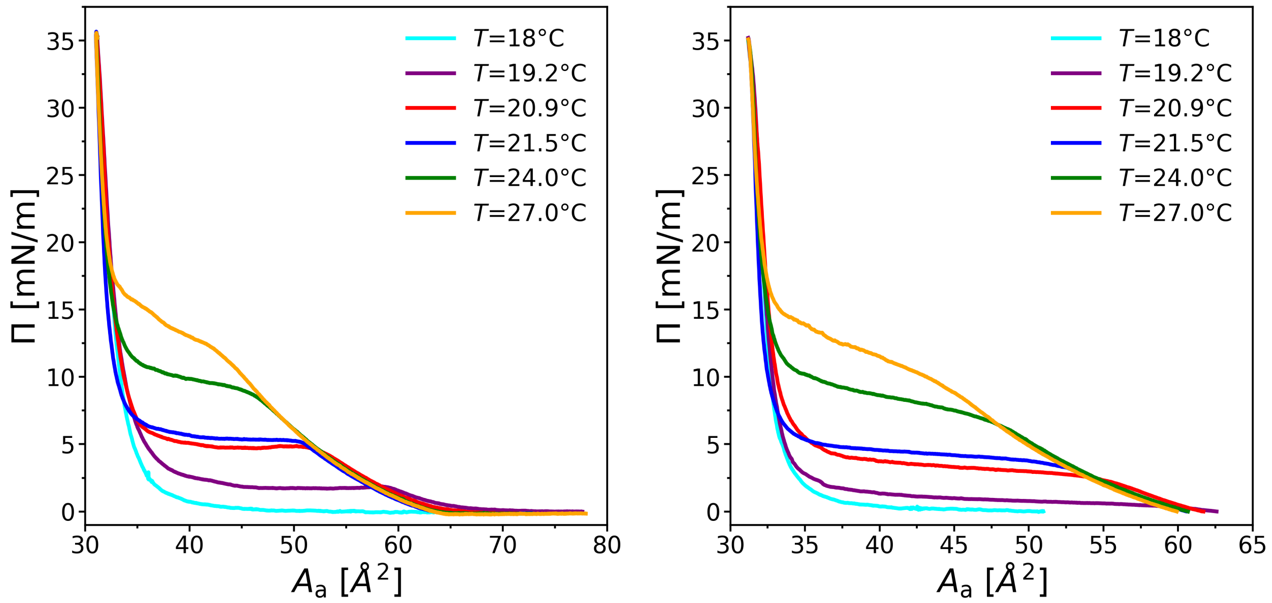

(a) (b)

Figure S2: Isotherm measurements of an D-enantiomer ($x_\mathrm{D} = 1$) monolayer on pH 3 water subphase on (a) compression and (b) decompression.

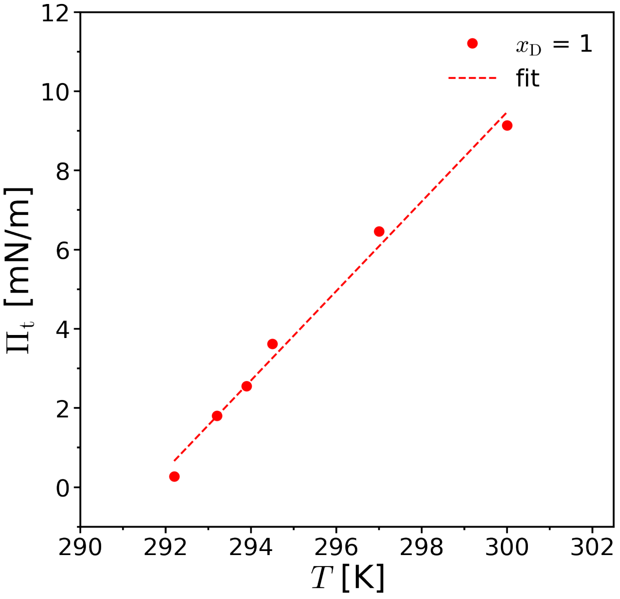

(a)

Figure S3: Temperature dependence of the main phase-transition pressure Π_t at the LE/LC phase transition of an D-enantiomer monolayer on pH 3 water subphase. Data for $T = 20.2$ °C were taken from.[1]

Table S1: Temperature (T) dependence of the transition pressure Π_t, Π_{LC}, pressure value at which the termination of the LE/LC transition occurs, the molecular area in the LC state (A_{LC}), the molecular area in the LE state (A_{LE}) of an D-enantiomer monolayer on pH 3 water subphase. Data for T = 20.2 °C were taken from.[1]

T [K]	T [°C]	Π_t [mN/m]	A_{LE} [Å^2]	Π_{LC} [mN/m]	A_{LC} [Å^2]	ΔA [Å^2]
292.2	19.2	0.3	60.4	1.6	33.6	-26.7
293.2	20.2	1.8				
293.9	20.9	2.6	54.5	4.3	33.5	-21.0
294.5	21.5	3.6	51.5	5.2	32.8	-18.7
297.0	24.0	6.5	48.0	10.8	32.9	-15.2
300.0	27.0	9.1	44.7	15.0	32.6	-12.1

1.2 D:L N-stearoyl-threonine mixed monolayer with $x_D = 0.9$

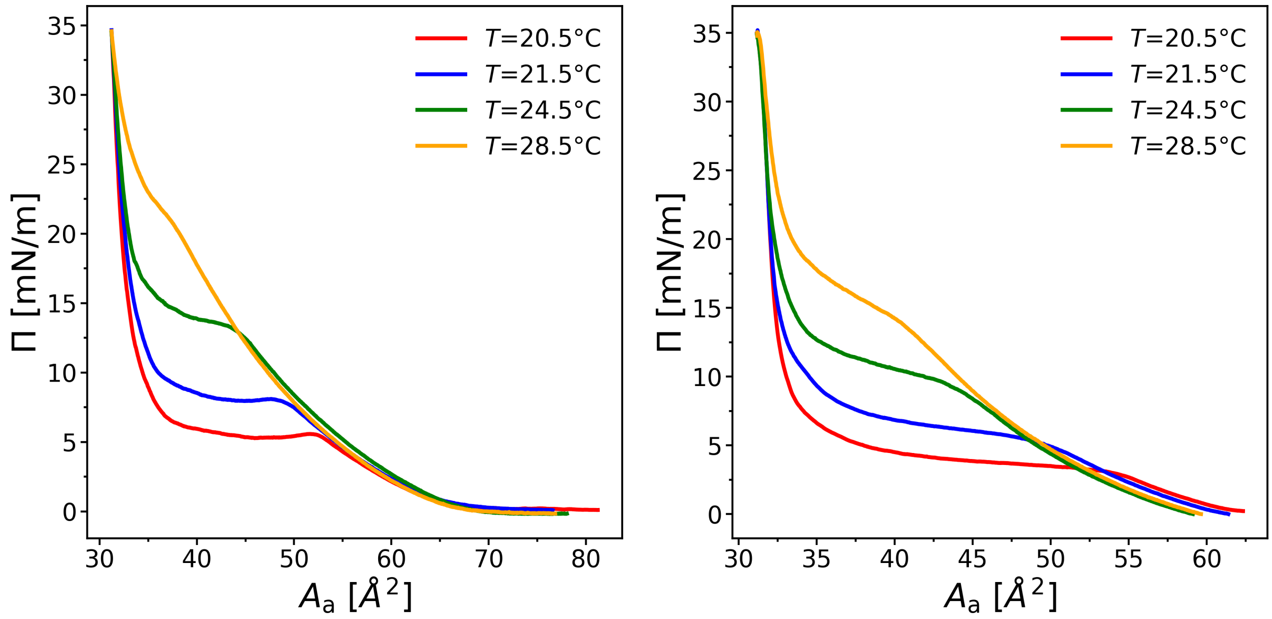

(a) (b)

Figure S4: Isotherm measurements of an D:L mixed monolayer with $x_D = 0.9$ on pH 3 water subphase on (a) compression and (b) decompression.

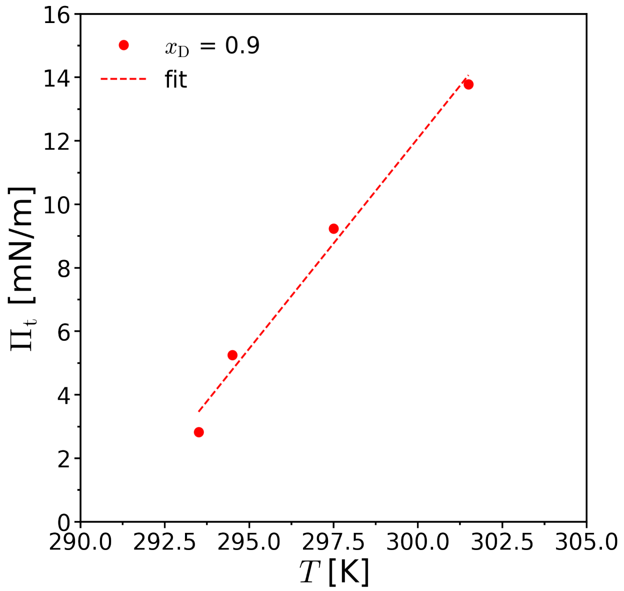

(a)

Figure S5: Temperature dependence of the main phase-transition pressure Π_t at the LE/LC phase transition of an D:L mixed monolayer with $x_D = 0.9$ on pH 3 water subphase.

Table S2: Temperature (T) dependence of the transition pressure Π_t, Π_{LC}, pressure value at which the termination of the LE/LC transition occurs, the molecular area in the LC state (A_{LC}), the molecular area in the LE state (A_{LE}) of an D:L mixed monolayer with $x_D = 0.9$ on pH 3 water subphase.

T [K]	T [°C]	Π_t [mN/m]	A_{LE} [Å^2]	Π_{LC} [mN/m]	A_{LC} [Å^2]	ΔA [Å^2]
293.5	20.5	2.8	54.2	5.5	33.0	-21.2
294.5	21.5	5.3	49.1	8.3	33.1	-16.0
297.5	24.5	9.2	43.9	12.9	33.2	-10.7
301.5	28.5	13.8	40.5	12.9	33.2	-7.3

1.3 D:L N-stearoyl-threonine mixed monolayer with $x_\mathrm{D} = 0.8$

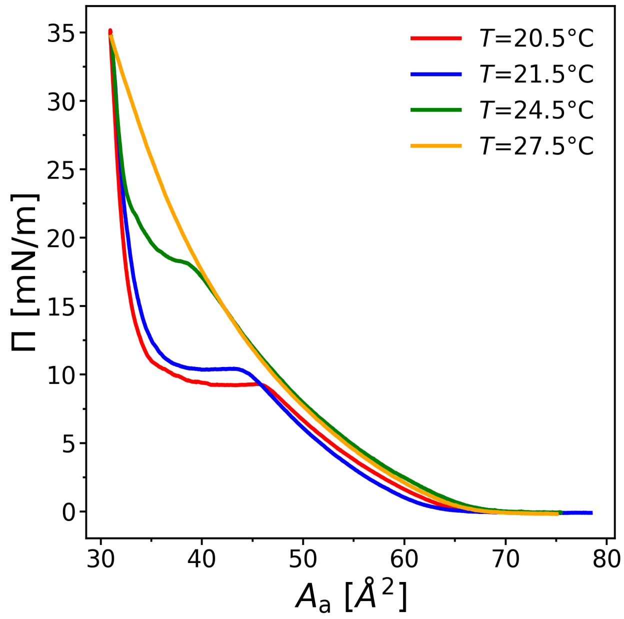

(a)

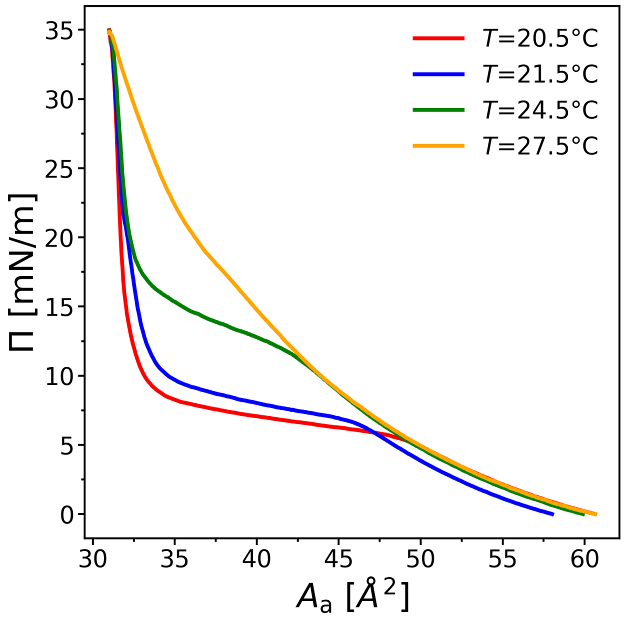

(b)

Figure S6: Isotherm measurements of an D:L mixed monolayer with $x_\mathrm{D} = 0.8$ on pH 3 water subphase on (a) compression and (b) decompression.

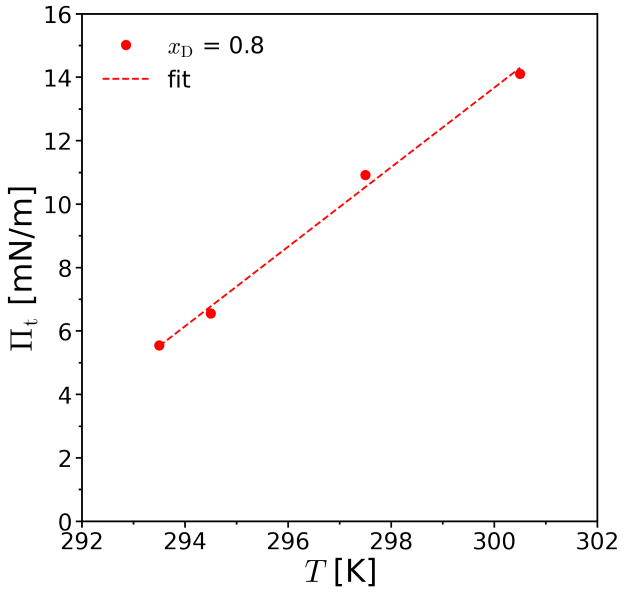

(a)

Figure S7: Temperature dependence of the main phase-transition pressure Π_t at the LE/LC phase transition of an D:L mixed monolayer with $x_\mathrm{D} = 0.8$ on pH 3 water subphase.

Table S3: Temperature (T) dependence of the transition pressure Π_t, Π_{LC}, pressure value at which the termination of the LE/LC transition occurs, the molecular area in the LC state (A_{LC}), the molecular area in the LE state (A_{LE}) for an D:L mixed monolayer with $x_D = 0.8$ on pH 3 water subphase.

T [K]	T [°C]	Π_t [mN/m]	A_{LE} [Å^2]	Π_{LC} [mN/m]	A_{LC} [Å^2]	ΔA [Å^2]
293.5	20.5	5.6	48.4	8.5	32.1	-16.2
294.5	21.5	6.6	45.8	9.9	32.7	-13.0
297.5	24.5	10.9	42.4	16.7	32.6	-9.8
300.5	27.5	14.1	36.8	21.5	33.0	-3.8

1.4 D:L N-stearoyl-threonine mixed monolayer with $x_D = 0.7$

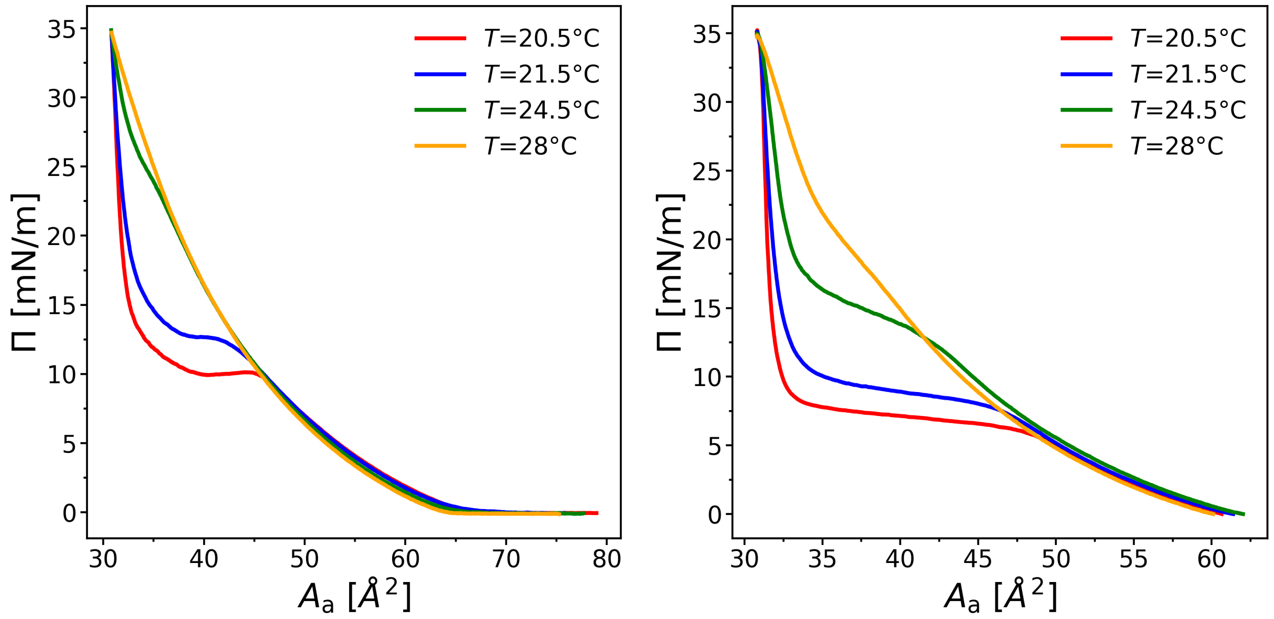

(a) (b)

Figure S8: Isotherm measurements of an D:L mixed monolayer with $x_D = 0.7$ on pH 3 water subphase on (a) compression and (b) decompression.

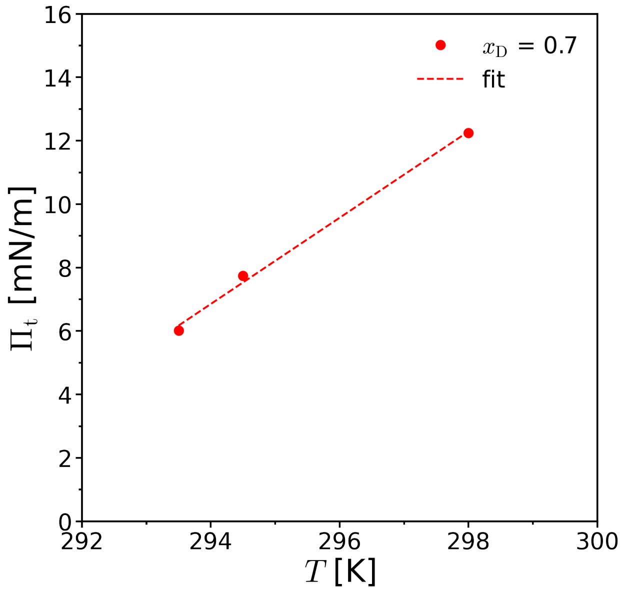

(a)

Figure S9: Temperature dependence of the main phase-transition pressure Π_t at the LE/LC phase transition of an D:L mixed monolayer with $x_D = 0.7$ on pH 3 water subphase.

Table S4: Temperature (T) dependence of the transition pressure Π_t, Π_{LC}, pressure value at which the termination of the LE/LC transition occurs, the molecular area in the LC state (A_{LC}), the molecular area in the LE state (A_{LE}) of an D:L mixed monolayer with $x_D = 0.7$ on pH 3 water subphase.

T [K]	T [°C]	Π_t [mN/m]	A_{LE} [Å^2]	Π_{LC} [mN/m]	A_{LC} [Å^2]	ΔA [Å^2]
293.5	20.5	6.0	48.0	8.2	32.2	-15.7
294.5	21.5	7.7	46.1	10.3	32.9	-13.2
298.0	25.0	12.2	42.4	17.5	33.2	-9.2

1.5 D:L N-stearoyl-threonine mixed monolayer with $x_{\mathrm{D}} = 0.6$

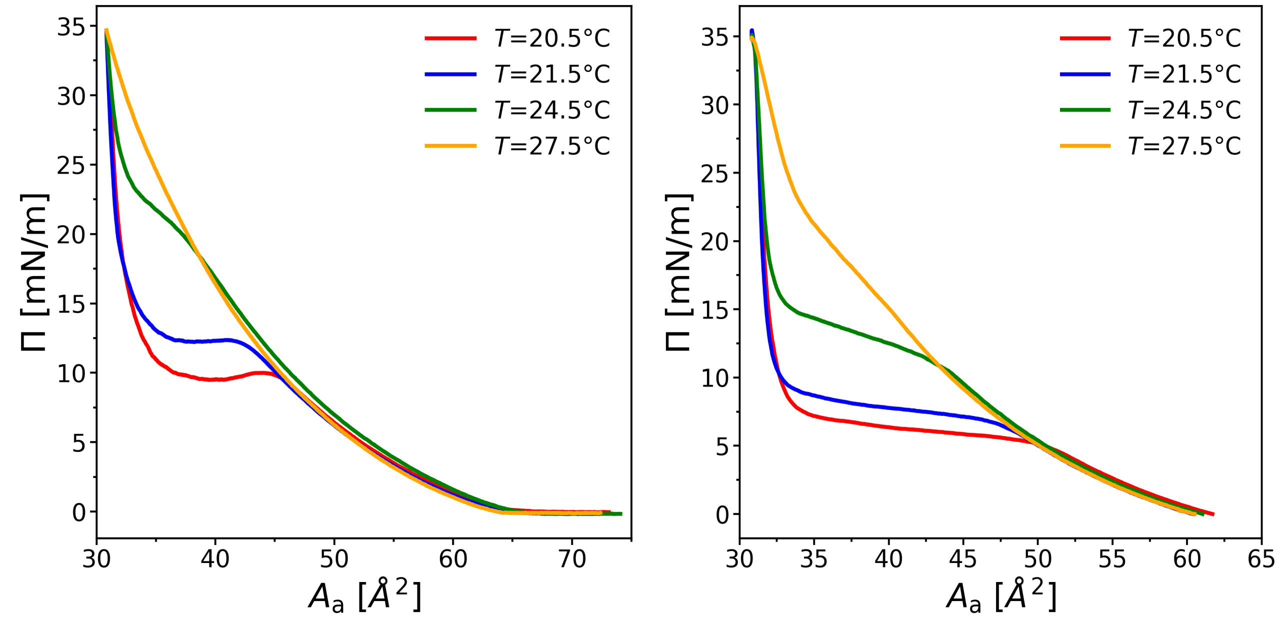

(a) (b)

Figure S10: Isotherm measurements of an D:L mixed monolayer with $x_{\mathrm{D}} = 0.6$ on pH 3 water subphase on (a) compression and (b) decompression.

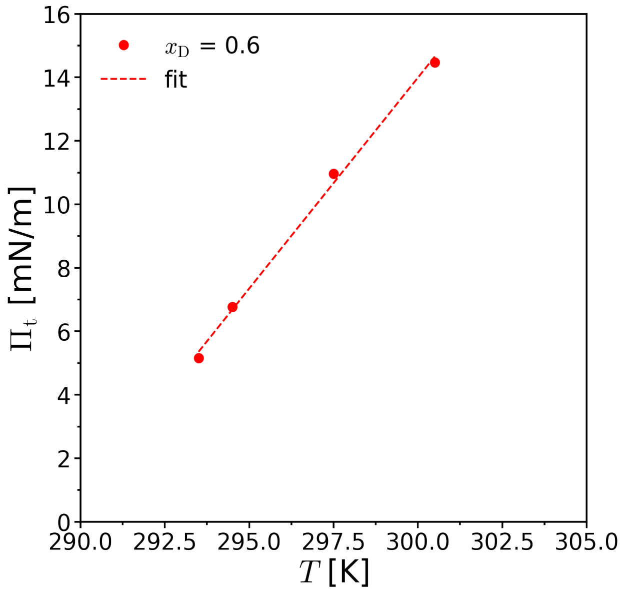

(a)

Figure S11: Temperature dependence of the main phase-transition pressure Π_{t} at the LE/LC phase transition of an D:L 6:4 mixed monolayer with $x_{\mathrm{D}} = 0.6$ on pH 3 water subphase.

Table S5: Temperature (T) dependence of the transition pressure Π_t, Π_{LC}, pressure value at which the termination of the LE/LC transition occurs, the molecular area in the LC state (A_{LC}), the molecular area in the LE state (A_{LE}) of an D:L mixed monolayer with $x_D = 0.6$ on pH 3 water subphase.

T [K]	T [°C]	Π_t [mN/m]	A_{LE} [Å^2]	Π_{LC} [mN/m]	A_{LC} [Å^2]	ΔA [Å^2]
293.5	20.5	5.2	50.2	7.4	32.9	-17.3
294.5	21.5	6.8	47.1	9.0	32.2	-14.8
297.5	24.5	11.0	43.3	15.5	32.6	-10.8
300.5	27.5	14.5	40.5	22.5	33.9	-6.6

1.6 D:L N-stearoyl-threonine mixed monolayer with $x_D = 0.5$

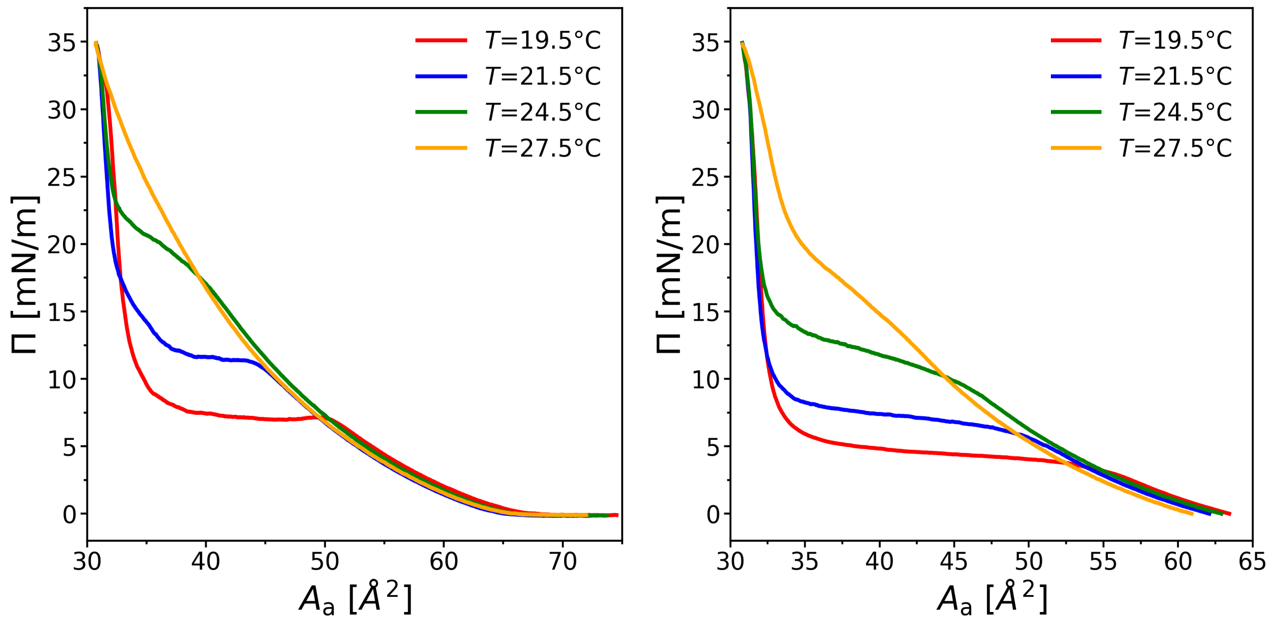

(a) (b)

Figure S12: Isotherm measurements of an D:L mixed monolayer with $x_D = 0.6$ on pH 3 water subphase on (a) compression and (b) decompression.

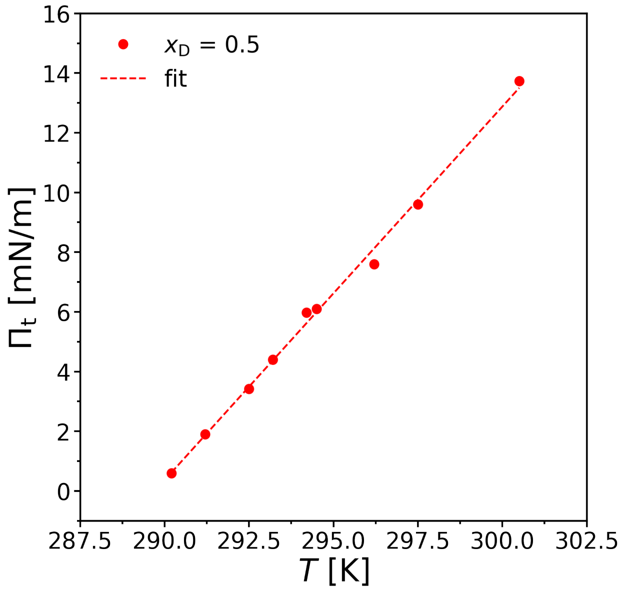

(a)

Figure S13: Temperature dependence of the main phase-transition pressure Π_t at the LE/LC phase transition of an D:L mixed monolayer with $x_D = 0.5$ on pH 3 water subphase. Data for $T = 17.2$, 18.2, 20.2, 21.2, 23.2°C were taken from.[1]

Table S6: Temperature (T) dependence of the transition pressure Π_t, Π_{LC}, pressure value at which the termination of the LE/LC transition occurs, the molecular area in the LC state (A_{LC}), the molecular area in the LE state (A_{LE}) of an D:L mixed monolayer with $x_D = 0.5$ on pH 3 water subphase. Data for T = 17.2, 18.2, 20.2, 21.2, 23.2°C were taken from.[1]

T [K]	T [°C]	Π_t [mN/m]	A_{LE} [Å^2]	Π_{LC} [mN/m]	A_{LC} [Å^2]	ΔA [Å^2]
290.2	17.2	0.6				
291.2	18.2	1.9				
292.5	19.5	3.4	54.2	5.9	33.0	-21.2
293.2	20.2	4.4				
294.2	21.2	6.1				
294.5	21.5	6.0	49.4	8.6	32.7	-16.8
296.2	23.2	7.6				
297.5	24.5	9.6	45.5	14.6	32.4	-13.1
300.5	27.5	13.7	41.1	14.6	34.0	-7.1

1.7 Temperature dependence of the main phase transition pressure Π_t

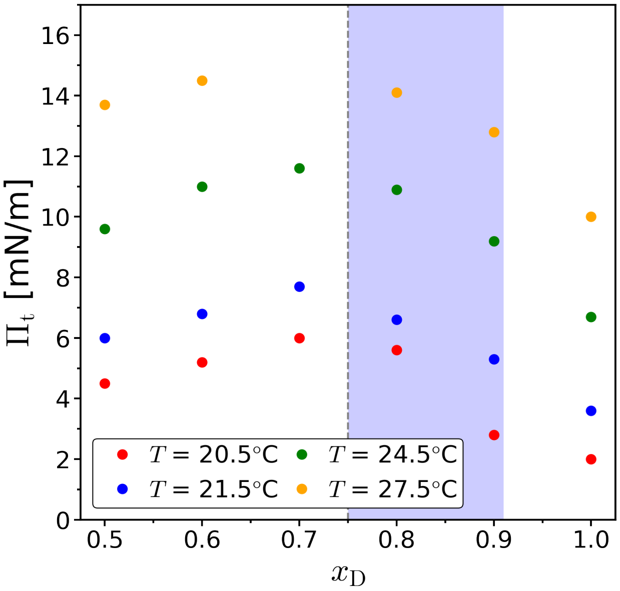

Figure S14: Variation of the transition pressure Π_t with the sample composition as a function of the sample temperature T.

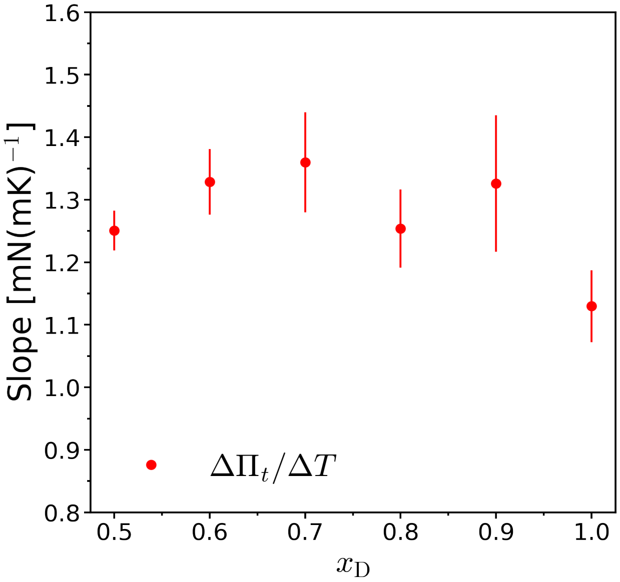

Figure S15: The variation of the slope $\Delta\Pi_t/\Delta T$ of the temperature dependence of the transition pressure with the sample composition.

Table S7: The estimated values of the T_0 and the slope $\Delta\Pi_t/\Delta T$ for the varied sample composition.

x_D	$T_0 \pm \Delta T_0$ [K]	$\Delta\Pi_t/\Delta T \pm \Delta(\Delta\Pi_t/\Delta T)$ [mN(mK)$^{-1}$]
1.0	291.7 ± 0.2	1.13 ± 0.06
0.9	291.0 ± 0.5	1.33 ± 0.11
0.8	289.1 ± 0.4	1.25 ± 0.06
0.7	289.0 ± 0.4	1.36 ± 0.08
0.6	289.5 ± 0.3	1.33 ± 0.05
0.5	289.7 ± 0.1	1.25 ± 0.03

2 GIXD

2.1 D:L N-stearoyl-threonine mixed monolayer with $x_\mathrm{D} = 0.7$

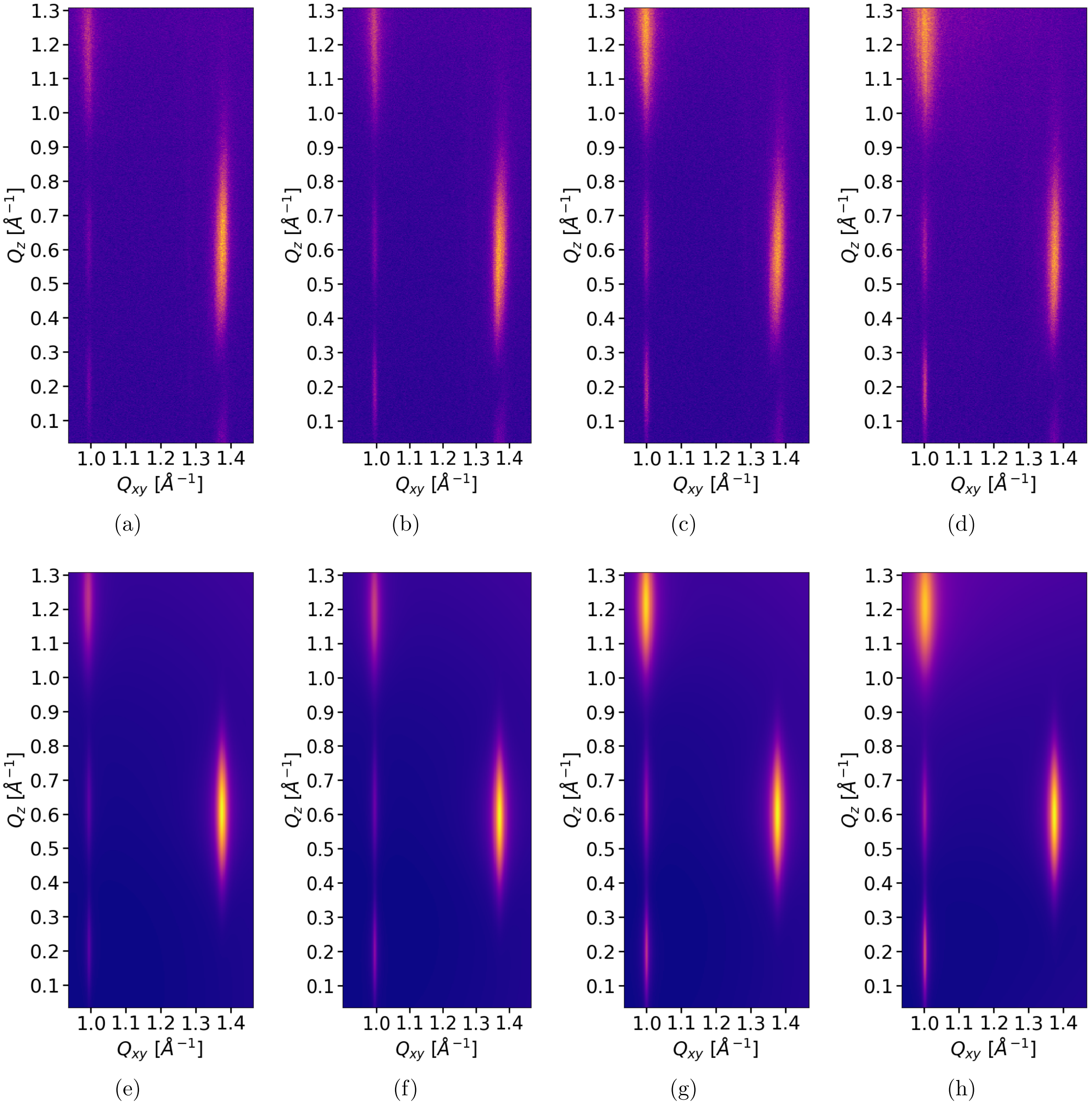

Figure S16: GIXD patterns (intensity vs. in-plane component Q_{xy} and out-of-plane component Q_z of the scattering vector Q) (top) and the corresponding fits (bottom) for an D:L mixed monolayer with $x_\mathrm{D} = 0.7$ at $\Pi = 10$ mN/m (a, e), $\Pi = 15$ mN/m (b, f), $\Pi = 25$ mN/m (c, g), and $\Pi = 35$ mN/m (d, h).

Table S8: GIXD data for an D:L mixed monolayer with $x_{\mathrm{D}} = 0.7$ at various pressures. Bragg peak positions Q^0_{xy} (± 0.003 Å^{-1}) and Q^0_z (± 0.005 Å^{-1}) and corresponding full-widths at half-maximum FWHM(w_{xy}) (± 0.003 Å^{-1}) and FWHM(w_z) (± 0.005 Å^{-1}) are presented.

Π [mN/m]	$Q^0_{xy}(1)$ [Å^2]	$Q^0_z(1)$ [Å^{-1}]	$Q^0_{xy}(2)$ [Å^{-1}]	$Q^0_z(2)$ [Å^{-1}]	$Q^0_{xy}(3)$ [Å^2]	$Q^0_z(3)$ [Å^{-1}]	$Q^0_{xy}(4)$ [Å^{-1}]	$Q^0_z(4)$ [Å^{-1}]	Data
10	0.991	1.229	0.991	0.212	0.991	0.636	1.376	0.614	Q^0
	0.03	0.294	0.011	0.206	0.014	0.247	0.027	0.294	FWHM
15	0.994	1.202	0.994	0.204	0.994	0.633	1.372	0.601	Q^0
	0.031	0.294	0.010	0.214	0.015	0.262	0.029	0.294	FWHM
25	0.998	1.215	0.998	0.202	0.998	0.633	1.377	0.608	Q^0
	0.036	0.294	0.012	0.215	0.016	0.256	0.03	0.294	FWHM
35	1.001	1.209	1.001	0.201	1.001	0.617	1.374	0.604	Q^0
	0.047	0.294	0.011	0.215	0.017	0.236	0.028	0.294	FWHM

Table S9: Structural parameters obtained from the GIXD data for an D:L mixed monolayer with $x_{\mathrm{D}} = 0.7$ at various pressures. Lattice parameters a, b, c (± 0.01 Å) and α, β, γ (± 0.1°), lattice distortion d (± 0.001), chain tilt t (± 0.1°) from the surface normal, in-plane area per alkyl chain A_{xy} (± 0.1 Å^2) and chain cross-sectional area A_0 (± 0.1 Å^2). The following constrains were applied: $Q^0_z(1) = Q^0_z(3)$*2.

Π [mN/m]	a, b, c [Å]	α, β, γ [°]	d	t [°]	A_{xy} [Å^2]	A_0 [Å^2]
10	4.89<br>6.80<br>6.80	137.8<br>111.1<br>111.1	0.3822	51.1	31.0	19.5
15	4.91<br>6.78<br>6.78	137.5<br>111.2<br>111.2	0.3764	50.4	31.1	19.8
25	4.89<br>6.76<br>6.76	137.5<br>111.2<br>111.2	0.3760	50.6	30.8	19.6
35	4.91<br>6.74<br>6.74	137.3<br>111.4<br>111.4	0.3708	50.4	30.8	19.6

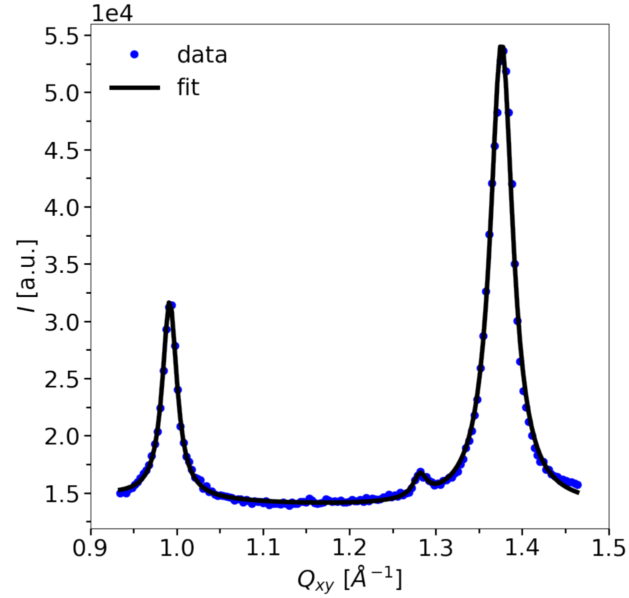

(a)

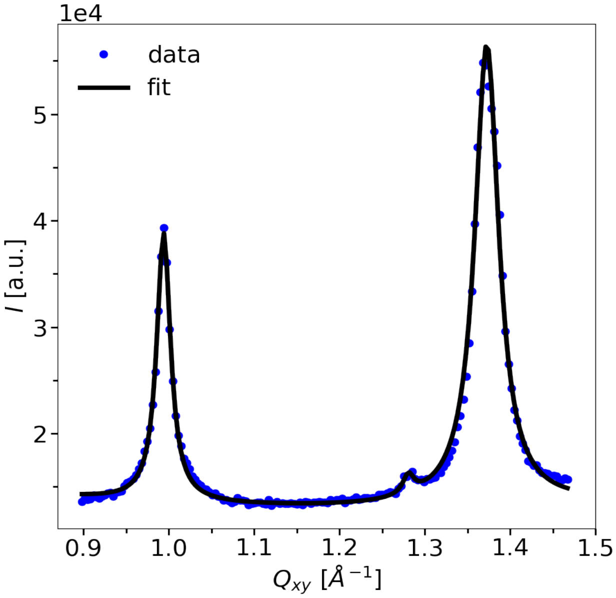

(b)

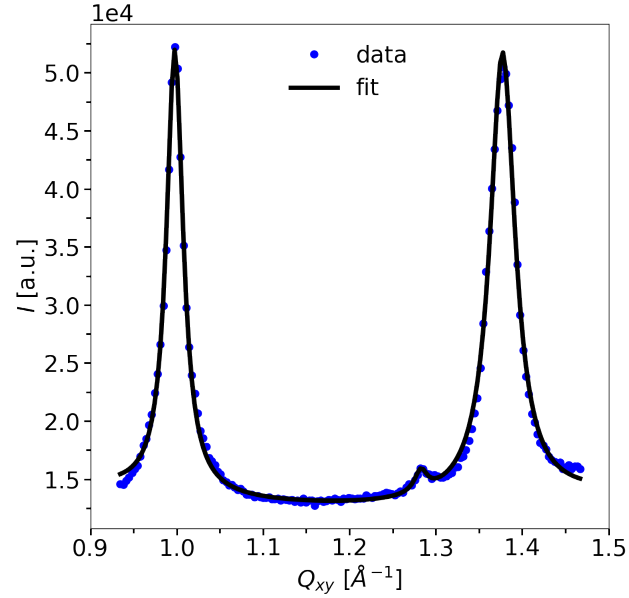

(c)

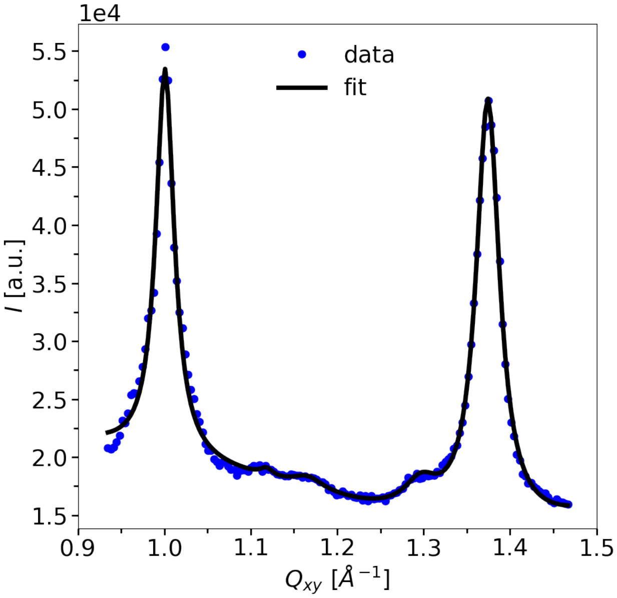

(d)

Figure S17: Q_z–integrated intensity (blue dots) vs. in-plane component Q_{xy} and the corresponding 1D fits (black lines) for an D:L mixed monolayer with $x_{\mathrm{D}} = 0.7$ at $\Pi = 10$ mN/m (a), $\Pi = 15$ mN/m (b), $\Pi = 25$ mN/m (c), and $\Pi = 35$ mN/m (d).

Table S10: GIXD data for an D:L mixed monolayer with x_D = 0.7 at various pressures obtained from the analysis of the Q_z–integrated intensity as a function of in-plane component Q_{xy}. Bragg peak positions Q^0_{xy} (± 0.003 Å^{-1}) and corresponding full-widths at half-maximum FWHM(w_{xy}) (± 0.003 Å^{-1}) are presented.

Π [mN/m]	$Q^0_{xy}(1)$ [Å^2]	$Q^0_{xy}(2)$ [Å^{-1}]	$Q^0_{xy}(3)$ [Å^{-1}]	$Q^0_{xy}(4)$ [Å^{-1}]	$Q^0_{xy}(5)$ [Å^2]	Data
10	0.992	1.282	1.376			Q^0
	0.020	0.019	0.032			FWHM
15	0.994	1.281	1.373			Q^0
	0.020	0.016	0.034			FWHM
25	0.998	1.283	1.377			Q^0
	0.023	0.018	0.035			FWHM
35	1.001	1.118	1.166	1.297	1.374	Q^0
	0.026	0.017	0.045	0.050	0.033	FWHM

2.2 Superlattice for an D:L mixed monolayer with $x_\mathbf{D}$ = 0.7

Table S11: Experimental and calculated GIXD data for an D:L mixed monolayer with x_D = 0.7 at 25 mN/m. Bragg peak positions Q^0_{xy} (± 0.003 Å^{-1}). Lattice parameters a, b, (± 0.01 Å), γ (± 0.1°). Miller indices h_s, k_s, lattice parameters a_s, b_s, and in-plane area A_crys (± 0.1 Å^2) of the corresponding super-lattice.

a, b, γ [$Å$, $Å$, °]	$Q^\mathrm{exp}_\mathrm{xy}$ [Å^{-1}]	$Q^\mathrm{cal}_\mathrm{xy}$ [Å^{-1}]	h_s, k_s	a_s, b_s [$Å$, $Å$]	γ_s [°]	A_crys [Å^2]
4.89, 6.76, 111.2	0.992	0.997	(0 -2), (0 2)	9.78	111.2	123.3
	1.282	1.285	(2 -1), (-2 1)	13.52		
	1.377	1.378	(2 0), (-2 0)			

2.3 D:L N-stearoyl-threonine mixed monolayer with $x_{\mathrm{D}} = 0.8$

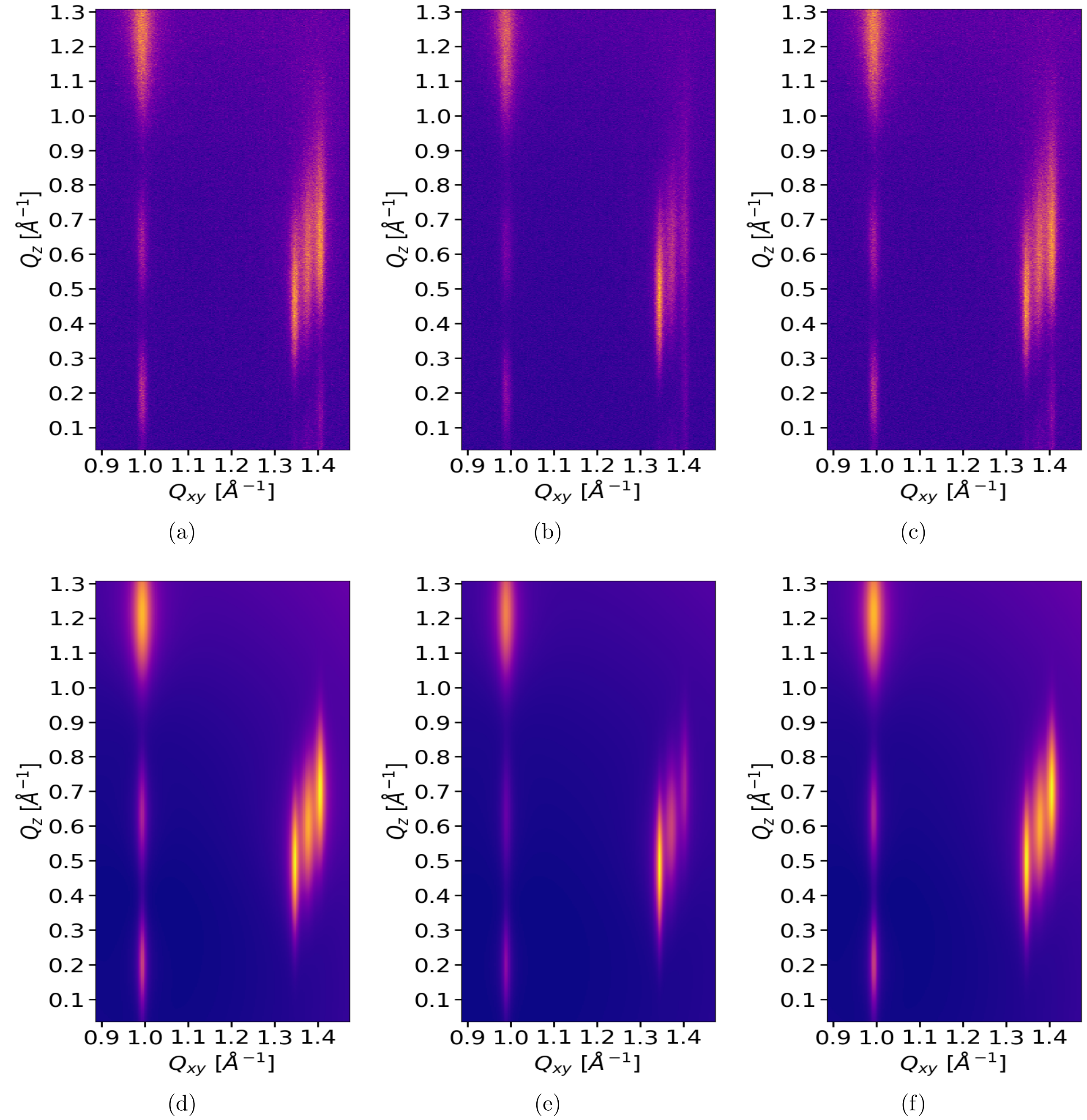

Figure S18: GIXD patterns (intensity vs. in-plane component Q_{xy} and out-of-plane component Q_z of the scattering vector Q) (top) and the corresponding fits (bottom) for an D:L mixed monolayer with $x_{\mathrm{D}} = 0.8$ at $\Pi = 10$ mN/m (a, d), $\Pi = 15$ mN/m (b, e), and $\Pi = 25$ mN/m (c, f).

Table S12: GIXD data for an D:L mixed monolayer with $x_{\mathrm{D}} = 0.8$ at various pressures. Bragg peak positions Q^0_{xy} (± 0.003 Å^{-1}) and Q^0_z (± 0.005 Å^{-1}) and corresponding full-widths at half-maximum FWHM(w_{xy}) (± 0.003 Å^{-1}) and FWHM(w_z) (± 0.005 Å^{-1}) are presented. The following constrains were applied: $Q^0_z(4) = Q^0_z(1)$ - $Q^0_z(2)$ and $Q^0_z(3) = Q^0_z(1)/2$.

Π [mN/m]	$Q^0_{xy}(1)$ [Å^2]	$Q^0_z(1)$ [Å^{-1}]	$Q^0_{xy}(2)$ [Å^{-1}]	$Q^0_z(2)$ [Å^{-1}]	$Q^0_{xy}(3)$ [Å^{-1}]	$Q^0_z(3)$ [Å^{-1}]	$Q^0_{xy}(4)$ [Å^{-1}]	$Q^0_z(4)$ [Å^{-1}]	$Q^0_{xy}(5)$ [Å^{-1}]	$Q^0_z(5)$ [Å^{-1}]	$Q^0_{xy}(6)$ [Å^{-1}]	$Q^0_z(6)$ [Å^{-1}]	Data
10	0.992<br>0.039	1.212<br>0.294	0.992<br>0.014	0.207<br>0.222	0.992<br>0.019	0.639<br>0.248	1.347<br>0.016	0.490<br>0.294	1.378<br>0.030	0.606<br>0.294	1.406<br>0.019	0.722<br>0.294	Q^0<br>FWHM
15	0.988<br>0.036	1.214<br>0.294	0.988<br>0.015	0.203<br>0.216	0.988<br>0.021	0.634<br>0.288	1.344<br>0.014	0.484<br>0.294	1.372<br>0.029	0.607<br>0.294	1.403<br>0.018	0.730<br>0.294	Q^0<br>FWHM
25	0.992<br>0.039	1.212<br>0.294	0.992<br>0.014	0.207<br>0.222	0.992<br>0.019	0.639<br>0.248	1.347<br>0.016	0.490<br>0.294	1.378<br>0.030	0.606<br>0.294	1.406<br>0.019	0.722<br>0.294	Q^0<br>FWHM

Table S13: Structural parameters obtained from the GIXD data for an D:L mixed monolayer with $x_{\mathrm{D}} = 0.8$ at various pressures. Lattice parameters a, b, c (± 0.01 Å) and α, β, γ (± 0.1°), lattice distortion d (± 0.001), chain tilt t (± 0.1°) from the surface normal, in-plane area per alkyl chain A_{xy} (± 0.1 Å^2) and chain cross-sectional area A_0 (± 0.1 Å^2).

Π [mN/m]	a, b, c [Å]	α, β, γ [°]	d	t [°]	A_{xy} [Å^2]	A_0 [Å^2]
10<br>Oblique	4.90<br>6.66<br>6.95	137.8<br>114.3<br>107.9	0.3863	50.7	31.1	19.7
10<br>Rectangular	4.89<br>6.79<br>6.79	137.8<br>111.1<br>111.1	0.3826	50.7	31.0	19.6
15<br>Oblique	4.91<br>6.68<br>6.97	137.9<br>114.2<br>107.9	0.3881	50.9	31.2	19.7
15<br>Rectangular	4.91<br>6.82<br>6.82	137.8<br>111.1<br>111.1	0.3823	50.9	31.2	19.7
25<br>Oblique	4.90<br>6.66<br>6.95	137.8<br>114.3<br>107.9	0.3863	50.7	31.1	19.7
25<br>Rectangular	4.88<br>6.79<br>6.79	137.8<br>111.1<br>111.1	0.3826	50.7	31.0	19.6

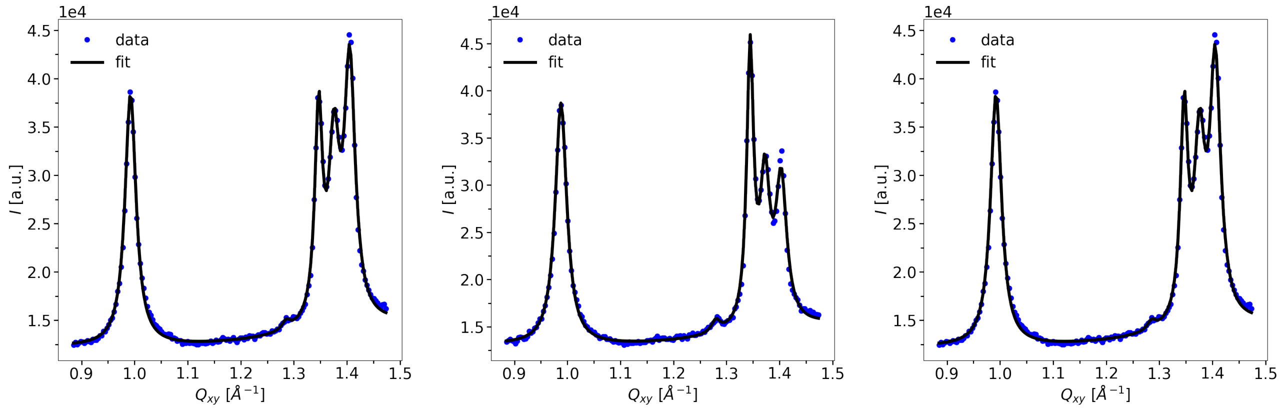

(a) (b) (c)

Figure S19: Q_z–integrated intensity (blue dots) vs. in-plane component Q_{xy} and the corresponding 1D fits (black lines) for an D:L mixed monolayer with x_D = 0.8 at Π = 10 mN/m (a), Π = 15 mN/m (b), Π = 25 mN/m (c).

Table S14: GIXD data for an D:L mixed monolayer with x_D = 0.8 at various pressures obtained from the analysis of the Q_z–integrated intensity as a function of in-plane component Q_{xy}. Bragg peak positions Q^0_{xy} (± 0.003 Å^{-1}) and corresponding full-widths at half-maximum FWHM(w_{xy}) (± 0.003 Å^{-1}) are presented.

Π [mN/m]	$Q^0_{xy}(1)$ [Å^2]	$Q^0_{xy}(2)$ [Å^{-1}]	$Q^0_{xy}(3)$ [Å^{-1}]	$Q^0_{xy}(4)$ [Å^{-1}]	$Q^0_{xy}(5)$ [Å^2]	Data
10	0.992	1.286	1.347	1.376	1.405	Q^0
	0.025	0.019	0.017	0.024	0.025	FWHM
15	0.988	1.280	1.345	1.373	1.403	Q^0
	0.027	0.020	0.015	0.022	0.025	FWHM
25	0.992	1.286	1.347	1.376	1.405	Q^0
	0.025	0.019	0.017	0.024	0.025	FWHM

2.4 Superlattice for an D:L mixed monolayer with $x_\mathbf{D} = 0.8$

Table S15: Experimental and calculated GIXD data for an D:L mixed monolayer with $x_\mathrm{D} = 0.8$. Bragg peak positions Q^0_{xy} (± 0.003 Å^{-1}). Lattice parameters a, b, (± 0.01 Å), γ (± 0.1°). Miller indices h_s, k_s, lattice parameters a_s, b_s, and in-plane area A_crys (± 0.1 Å^2) of the corresponding super-lattice.

a, b, γ [Å, Å, °]	$Q^{\mathrm{exp}}_{\mathrm{xy}}$ [Å^{-1}]	$Q^{\mathrm{cal}}_{\mathrm{xy}}$ [Å^{-1}]	h_s, k_s	a_s, b_s [Å, Å]	γ_s [°]	A_crys [Å^2]
4.90, 6.66, 107.9	0.992	0.991	(0 -2), (0 2)	9.8	107.9	124.2
(oblique)	1.286	1.285	(2 -1), (-2 1)	13.32		
	1.347	1.348	(2 0), (-2 0)			
	1.406	1.406	(-2 2), (2 -2)			
4.88, 6.79, 111.1	0.992	0.991	(0 -2), (0 2)	9.76	111.1	123.7
(rectangular)	1.286	1.288	(2 -1), (-2 1)	13.58		
	1.378	1.379	(2 -2), (-2 2)			

2.5 D:L N-stearoyl-threonine mixed monolayer with $x_\mathrm{L} = 0.9$

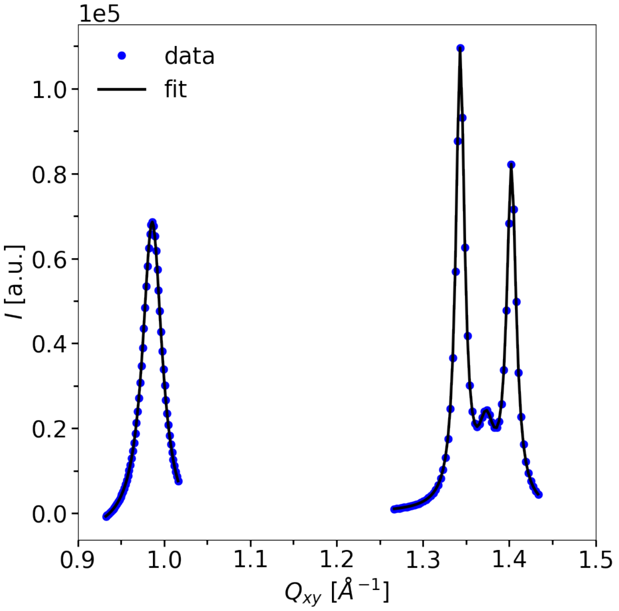

(a)

Figure S20: Q_z–integrated intensity (blue dots) vs. in-plane component Q_{xy} and the corresponding fits (black lines) for an D:L mixed monolayer with $x_\mathrm{L} = 0.9$ at $\Pi = 10$ mN/m.

Table S16: GIXD data for an D:L mixed monolayer with $x_{\mathrm{L}} = 0.9$ at various pressures. Bragg peak positions Q_{xy}^0 ($\pm$ 0.003 Å^{-1}) and Q_z^0 ($\pm$ 0.01 Å^{-1}) and corresponding full-widths at half-maximum FWHM(w_{xy}) ($\pm$ 0.003 Å^{-1}) and FWHM(w_z) ($\pm$ 0.01 Å^{-1}) are presented. The following constrains were applied: $Q_z^0(4) = Q_z^0(1)$ - $Q_z^0(2)$. The Q_z data of the rectangular unit cell are not fitted because of low intensity and strong overlapping with the data of the oblique lattice.

Π [mN/m]	$Q_{xy}^0(1)$ [Å^2]	$Q_z^0(1)$ [Å^{-1}]	$Q_{xy}^0(2)$ [Å^{-1}]	$Q_z^0(2)$ [Å^{-1}]	$Q_{xy}^0(3)$ [Å^{-1}]	$Q_{xy}^0(4)$ [Å^{-1}]	$Q_z^0(4)$ [Å^{-1}]	Data
5	0.993	1.19	1.341	0.50	1.371	1.400	0.69	Q^0
	0.021	0.28	0.012	0.28	0.024	0.016	0.28	FWHM
10	0.991	1.20	1.343	0.49	1.373	1.403	0.71	Q^0
	0.014	0.28	0.012	0.28	0.025	0.013	0.28	FWHM
15	0.993	1.21	1.343	0.49	1.374	1.405	0.72	Q^0
	0.023	0.28	0.012	0.28	0.022	0.015	0.28	FWHM
20	0.996	1.16	1.345	0.48	1.373	1.407	0.68	Q^0
	0.031	0.28	0.012	0.28	0.023	0.018	0.28	FWHM
25	0.992	1.15	1.346	0.48	1.372	1.405	0.67	Q^0
	0.040	0.28	0.010	0.28	0.028	0.016	0.28	FWHM
30	1.004	1.15	1.346	0.47	1.370	1.409	0.68	Q^0
	0.042	0.28	0.012	0.28	0.026	0.021	0.28	FWHM

Table S17: Structural parameters obtained from the GIXD data for an D:L mixed monolayer with $x_{\mathrm{L}} = 0.9$ at various pressures. Lattice parameters a, b, c (± 0.01 Å) and α, β, γ (± 0.1°), lattice distortion d (± 0.001), chain tilt t (± 0.3°) from the surface normal, in-plane area per alkyl chain A_{xy} (± 0.1 Å^2) and chain cross-sectional area A_0 (± 0.1 Å^2).

Π [mN/m]	a, b, c [Å]	α, β, γ [°]	d	t [°]	A_{xy} [Å^2]	A_0 [Å^2]
5<br>Oblique	4.93<br>6.65<br>6.95	137.6<br>114.4<br>108.0	0.3811	50.2	31.2	20.0
10<br>Oblique	4.92<br>6.66<br>6.96	137.8<br>114.4<br>107.9	0.3850	50.4	31.2	19.8
15<br>Oblique	4.91<br>6.65<br>6.95	137.7<br>114.5<br>107.8	0.3841	50.6	31.1	19.7
20<br>Oblique	4.91<br>6.63<br>6.93	137.6<br>114.5<br>107.9	0.3825	49.4	31.0	20.2
25<br>Oblique	4.91<br>6.66<br>6.95	137.8<br>114.3<br>107.9	0.3856	49.2	31.1	20.3
30<br>Oblique	4.91<br>6.58<br>6.89	137.3<br>114.7<br>108.0	0.3759	48.9	30.7	20.2

2.6 D:L N-stearoyl-threonine mixed monolayer with $x_{\mathrm{D}} = 0.5$

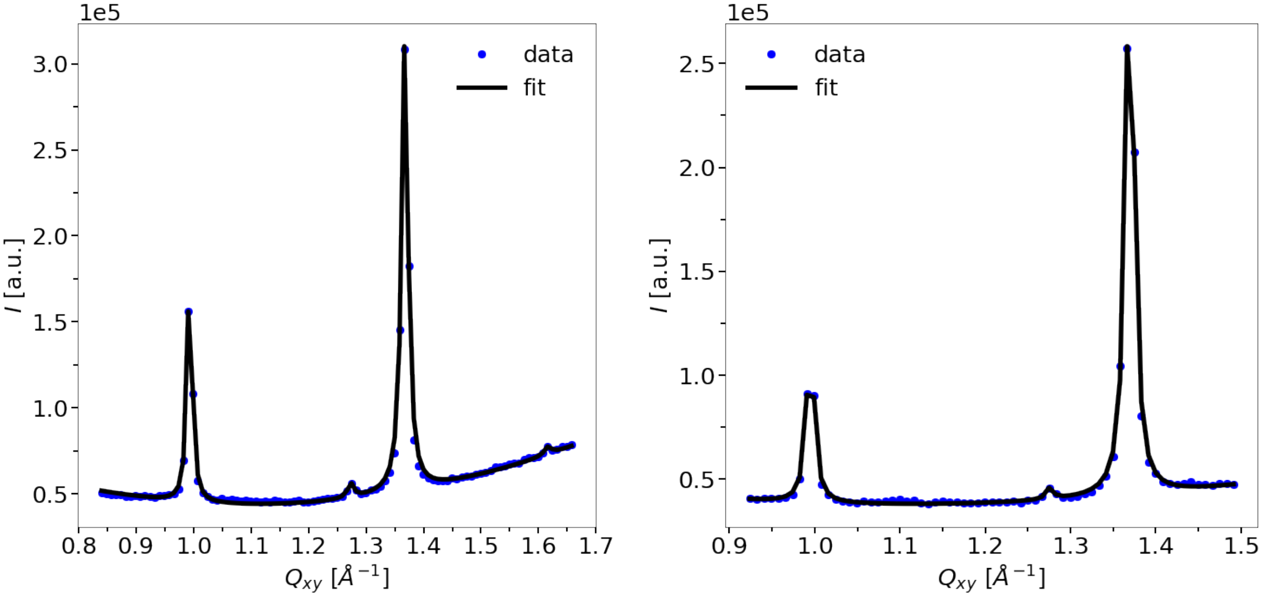

(a) (b)

Figure S21: Q_z–integrated intensity (blue dots) vs. in-plane component Q_{xy} and the corresponding 1D fits (black lines) for an D:L mixed monolayer with $x_{\mathrm{D}} = 0.5$ at $\Pi = 5$ mN/m (a) and $\Pi = 10$ mN/m (b).

Table S18: GIXD data for an D:L mixed monolayer with $x_{\mathrm{D}} = 0.5$ obtained from the analysis of the Q_z–integrated intensity as a function of in-plane component Q_{xy}. Bragg peak positions Q^0_{xy} ($\pm$ 0.003 Å^{-1}) and corresponding full-widths at half-maximum FWHM(w_{xy}) ($\pm$ 0.003 Å^{-1}) are presented.

Π [mN/m]	$Q^0_{xy}(1)$ [Å^2]	$Q^0_{xy}(2)$ [Å^{-1}]	$Q^0_{xy}(3)$ [Å^{-1}]	$Q^0_{xy}(4)$ [Å^2]	Data
5	0.994	1.273	1.368	1.616	Q^0
	0.010	0.013	0.013	0.015	FWHM
10	0.995	1.276	1.37		Q^0
	0.010	0.010	0.011		FWHM

2.7 N-stearoyl-L-threonine monolayer

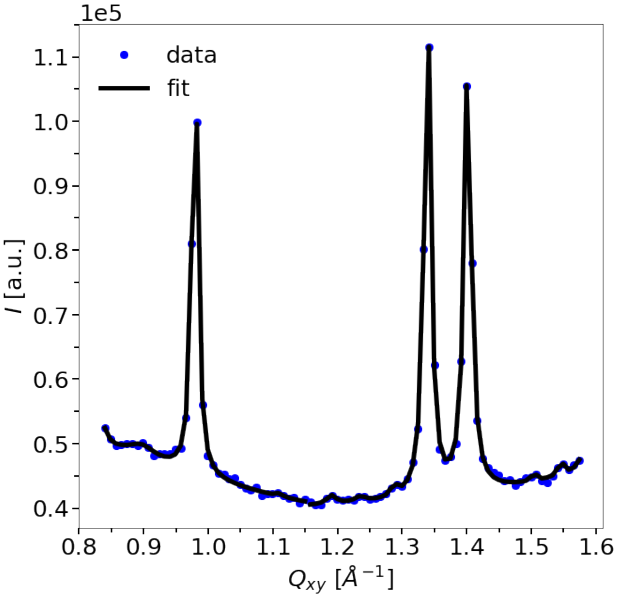

Figure S22: Q_z–integrated intensity (blue dots) vs. in-plane component Q_{xy} and the corresponding fits (black lines) for an N-stearoyl-L-threonine monolayer at $\Pi = 10$ mN/m.

Table S19: GIXD data for an N-stearoyl-L-threonine monolayer at $\Pi = 10$ mN/m obtained from the analysis of the Q_z–integrated intensity as a function of in-plane component Q_{xy}. Bragg peak positions Q_{xy}^0 ($\pm$ 0.003 Å^{-1}) and corresponding full-widths at half-maximum FWHM(w_{xy}) ($\pm$ 0.003 Å^{-1}) are presented.

Π [mN/m]	$Q_{xy}^0(1)$ [Å^2]	$Q_{xy}^0(2)$ [Å^{-1}]	$Q_{xy}^0(3)$ [Å^{-1}]	$Q_{xy}^0(4)$ [Å^2]	$Q_{xy}^0(5)$ [Å^2]	$Q_{xy}^0(6)$ [Å^{-1}]	$Q_{xy}^0(7)$ [Å^{-1}]	$Q_{xy}^0(8)$ [Å^2]	$Q_{xy}^0(9)$ [Å^2]	$Q_{xy}^0(10)$ [Å^{-1}]	$Q_{xy}^0(11)$ [Å^{-1}]	$Q_{xy}^0(12)$ [Å^{-1}]	Data
10	0.841	0.897	0.979	1.110	1.191	1.235	1.288	1.339	1.402	1.504	1.546	1.590	Q^0
	0.010	0.050	0.010	0.010	0.018	0.021	0.012	0.012	0.013	0.005	0.010	0.015	FWHM

2.8 GIXD data analysis

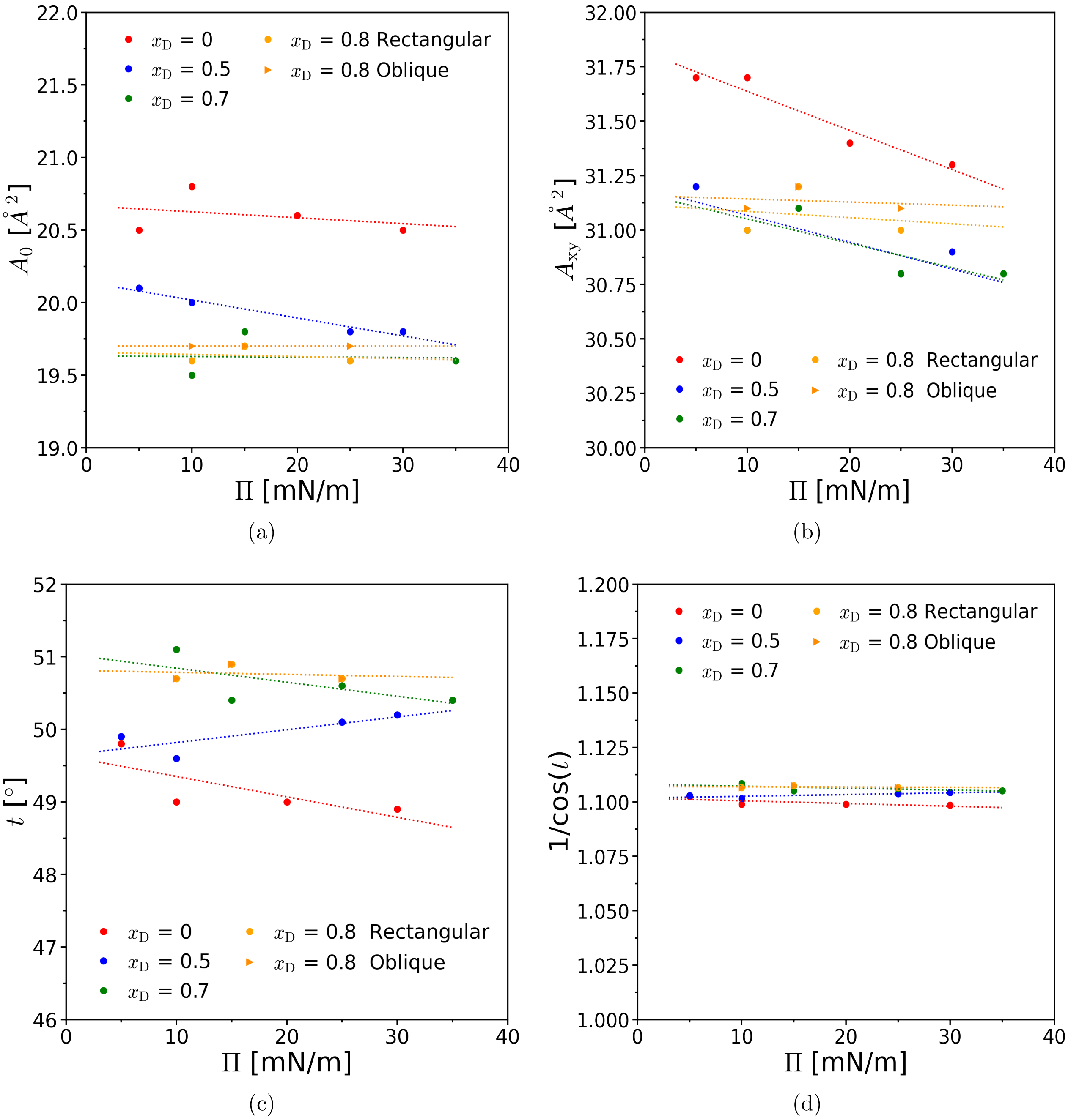

Figure S23: Pressure dependence of (a) the chain cross-sectional area (A_0), (b) in plane area per alkyl chain (A_{xy}), (c) tilt angle (t) and (d) 1/cos(t). Data for L-enantiomer and D:L racemate were taken from[1] and were measured at $T = 10$ °C. Data for the D:L mixed monolayers with $x_D = 0.7$ and with $x_D = 0.8$ were collected at $T = 20$ °C.

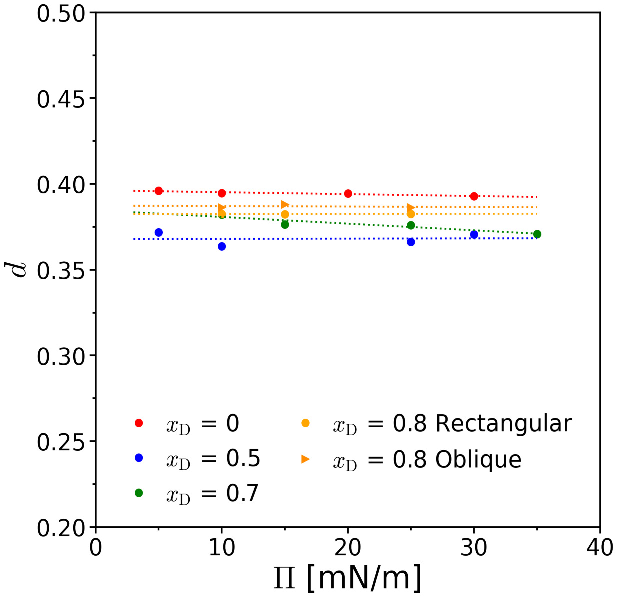

Figure S24: Pressure dependence of the distortion (d). Data for L-enantiomer and D:L racemate were taken from[1] and were measured at $T = 10°C$. Data for the D:L mixed monolayers with $x_D = 0.7$ and with $x_D = 0.8$ were collected at $T = 20°C$.

References

(1) Vollhardt, D.; Stefaniu, C.; Brezesinski, G. Special features of monolayer characteristics of N-alkanoyl substituted threonine amphiphiles. Physical Chemistry Chemical Physics **2019**, 21, 96–103.
